# A cloud-based miniscope for neurosurveillance of brain health and disease in freely behaving animals

**DOI:** 10.1038/s41592-026-03111-z

**Published:** 2026-06-22

**Authors:** Janaka Senarathna, Darren Yang, Julia Brill, Subhrajit Das, Shruthi Bare, Yunke Ren, Devorah VanNess, Vu Dinh, Irfaan Karim, Amit K. Banerjee, Nitish V. Thakor, Mingyao Ying, David J. Linden, Arvind P. Pathak

**Affiliations:** 1 The Russell H. Morgan Department of Radiology and Radiological Science, The Johns Hopkins University School of Medicine, Baltimore, MD USA; 2 Chemical and Biomolecular Engineering, Johns Hopkins University, Baltimore, MD USA; 3 The Solomon H. Snyder Department of Neuroscience, The Johns Hopkins University School of Medicine, Baltimore, MD USA; 4 Electrical and Computer Engineering, Johns Hopkins University, Baltimore, MD USA; 5 Biomedical Engineering, The Johns Hopkins University School of Medicine, Baltimore, MD USA; 6 Neurology, The Johns Hopkins University School of Medicine, Baltimore, MD USA; 7 The Kennedy Krieger Institute, Baltimore, MD USA; 8 Applied Physics Lab, Johns Hopkins University, Baltimore, MD USA; 9 The Sidney Kimmel Comprehensive Cancer Center, Baltimore, MD USA

**Keywords:** Neuro-vascular interactions, Cancer imaging, Cancer microenvironment, Neurophysiology, Preclinical research

## Abstract

Miniaturized microscopes or ‘miniscopes’ for neuroimaging in freely behaving animals mostly operate over short durations (<2 h) and image either neuronal activity or cerebral hemodynamics. In contrast, central nervous system (CNS) disease models involving seizures, brain tumors etc. necessitate long-term (>24 h) imaging, remote operation and simultaneous characterization of multiple neurophysiological variables such as neuronal activity, blood flow, blood volume, oxygenation and cellular dynamics (a capability that we call ‘neurosurveillance’). Thus, we developed the ‘CloudScope’, a cloud-based multicontrast miniscope for autonomous neurosurveillance in freely behaving animals. Its cloud-based architecture enables global remote operation and continuous acquisition of multicontrast images over CNS disease model life cycles. We demonstrate CloudScope’s neurosurveillance capabilities in predicting behavior from 24-h neuroimaging data with deep learning (DL), characterizing neurovascular changes during natural behavior, seizure-induced neurovascular disruptions, and in vivo cellular and microvascular phenotyping of brain tumor microenvironments. Finally, CloudScope’s architecture enables ‘time-shared’ imaging, which potentially reduces animal use. Collectively, CloudScope’s neurosurveillance capabilities in conjunction with CNS disease models establish a new paradigm for characterizing their etiology and evolution.

## Abstract (web summary)

A miniscope with cloud-based operation and multicontrast capabilities enables continuous recording of neuronal activity, hemodynamics or cellular dynamics over multiple days. This allows monitoring brain function in neurological disease models for example undergoing seizures or harboring brain tumors.

## Main

The ability to conduct continuous (≥24 h), neuroimaging of multiple physiological variables (for example neuronal activity (Neu_ACT_), blood flow, blood volume, oxygenation and cellular dynamics) within the CNS microenvironment is crucial for characterizing preclinical models of CNS disease (Supplementary Table 1). We refer to this continuous multimodality neuroimaging capability as ‘neurosurveillance’^1^. Neurosurveillance can empower us to elucidate the etiology, progression and therapeutic response of CNS diseases ranging from seizures to brain cancer, while simultaneously permitting their correlation with behavioral outcomes. Recent advances in miniaturized optical microscopes (‘miniscopes’^2^), have made neuroimaging in freely behaving, unrestrained animals a reality; however, most extant miniscopes are specifically designed to interrogate healthy brain function and acquire images with a single image contrast mechanism based on Neu_ACT_^2^ or vascular function^3^, but usually not both, at a high frame rate (for example 10–20 Hz) over short durations (for example <2 h) (Supplementary Table 2). This short-duration, single-modality imaging approach limits the utility of most miniscopes for conducting neurosurveillance in preclinical models of CNS disease because many pathological changes occur over substantially longer timescales (for example >24 h to days), and often involve the dysregulation of both neuronal and vascular function. Moreover, technical hurdles such as the lack of an architecture for remote miniscope operation to enable continuous imaging for ≥24 h; cloud-based image streaming to enable operators to check image acquisition in real-time; photobleaching; image sensor overheating; a need for proprietary electronic and optical parts; and limited battery life, have made it challenging to conduct neurosurveillance of CNS disease models. To circumvent these limitations, we developed a miniscope system called the CloudScope, which integrates a three-dimensional (3D)-printed miniscope designed for long-term multicontrast neuroimaging with an internet of things (IoT) networked architecture. The CloudScope can track Neu_ACT_, cerebral blood flow (CBF), cerebral blood volume (CBV), intravascular oxygenation (Hb_SAT_) and cells in vivo for neurosurveillance in freely behaving animals. CloudScope was implemented using inexpensive, commercially available, hobbyist or open-source components to encourage ubiquitous usage and enhancement by the broader neuroscientific community. Once the CloudScope has been mounted on an animal, its cloud-based architecture and image-acquisition scheme enable continuous multicontrast imaging for ≥24 h without sensor overheating or photobleaching, while permitting in vivo neuroimaging from anywhere in the world. We demonstrate the widespread potential of CloudScope via a host of continuous (≥24 h) neurosurveillance applications in healthy animals and CNS disease models that include: (1) predicting animal behavior from continuously acquired Neu_ACT_ data with deep learning (DL); (2) characterizing neurovascular changes in the brains of freely behaving healthy mice; (3) assessing seizure-induced disruptions and subsequent recovery of Neu_ACT_ and neurovascular function; and (4) an in vivo assay for cellular and vascular phenotyping during brain tumor evolution. We believe these innovations and their broad applicability make the CloudScope a powerful tool for characterizing long-term, in vivo changes in the CNS microenvironment in health and disease.

## Results

### CloudScope enabled remote neurosurveillance over 24 h in healthy freely behaving animals

**Fig. 1:**
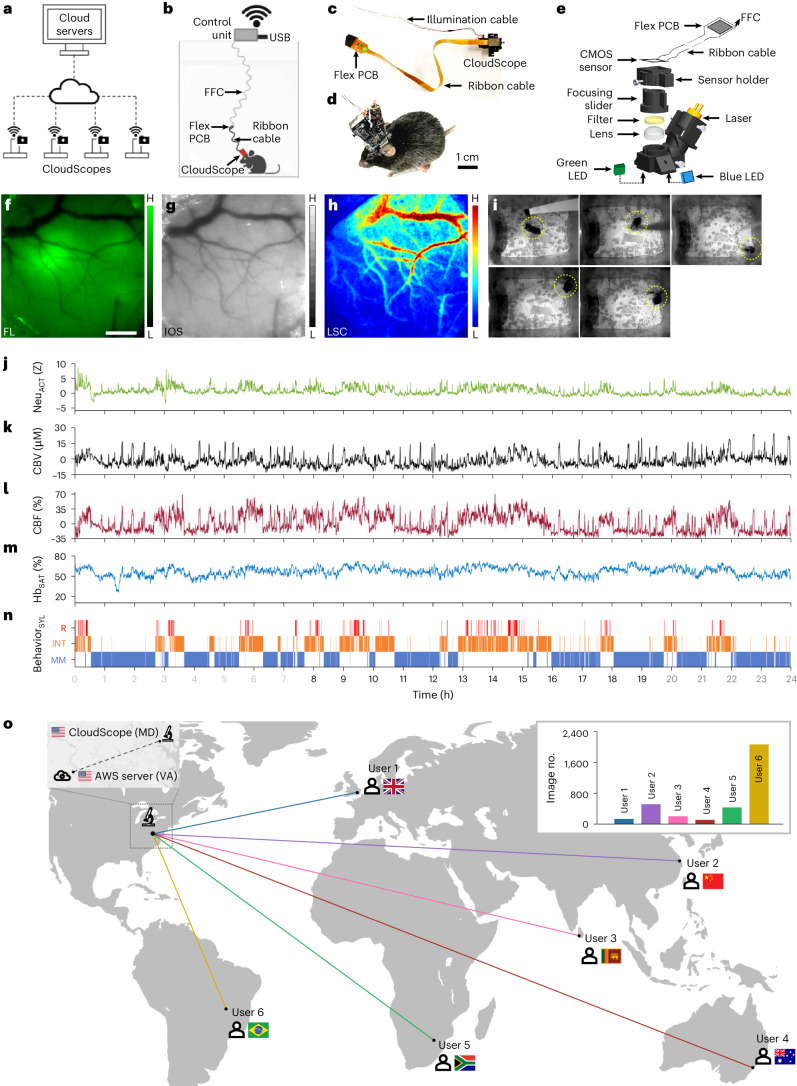
CloudScope enabled remote neurosurveillance over 24 h in healthy freely behaving animals. **a**, Schematic illustrating the cloud-based IoT architecture that supports internet access to multiple CloudScopes. CloudScopes can be housed in a laboratory or an animal facility and connect to a cloud-based server via local Wi-Fi. **b**, Schematic of the system setup. A control unit placed above the cage handled all communications with the cloud-based server. A USB attached to the control unit enabled local image storage. The control unit was linked to the CloudScope via a 1 m FFC, a flex PCB integrated with a 30-cm ribbon cable, for power and image transfer. An additional set of thin 32 G (200-μm diameter) wires carried power from the control unit to the CloudScope’s illumination sources. **c**, A photograph of the CloudScope, showing the 30-cm ribbon cable and integrated flex PCB, and 32 G wires. **d**, Photograph of the CloudScope mounted on a freely behaving mouse. **e**, Schematic illustrating key optical components of the CloudScope. Blue and green LEDs (453/530 ± 20 nm), a laser diode (670 nm or 680 nm), a fixed focus lens (lens, *f* = 4.6 mm) and a long-pass filter (cutoff of 510 nm) for imaging green FL, IOS and LSC over the same cortical FoV. Image focus was achieved by manually adjusting the linear focusing slider, which was then locked in place with set screws. **f**–**h**, Maps of neuronal calcium dynamics (via FL of GCaMP6s-bound Ca^2+^ in neuropil or neuronal soma) (**f**); microvascular architecture (via IOS) (**g**); and CBF (via LSC) (**h**) from a 3 × 3 mm^2^ FoV in the mouse cortex (center = −2/+2 mm AP/ML with respect to the bregma) simultaneously acquired with the CloudScope. **i**, Animal behavior was captured via a separate behavioral camera that was synchronized with multicontrast neuroimaging. **j**–**n**, A 24-h time-series of Neu_ACT_ (**j**); CBV (**k**); CBF (**l**); intravascular Hb_SAT_ (**m**); and the animal’s behavioral state (**n**) recorded from a freely behaving mouse. Neuroimaging time-series shown are spatial averages from the parenchyma (excluding surface vessels) and denoised with a 30-s median filter for visualization purposes. The behavior of the animal was classified as belonging to one of three Behavior_SYL_ (MM, INT or R). The light–dark cycle is denoted by gray–black time axis labels. **o**, A world map illustrating the locations from which the CloudScope was remotely accessed. These remote access points included UK (London), China (Ningbo), Sri Lanka (Colombo), Australia (Sydney), South Africa (Pretoria) and Brazil (Brasilia). Users from these locations are labeled as User 1…, User 6, respectively, with pseudocolored traces indicating CloudScope access. Inset on the top-right shows the number of images acquired by each user (note, users acquired images of a test pattern to ensure compliance with experimentation rules in the participating countries). As highlighted by the inset on the top-left, users gained access to the CloudScope housed in Baltimore, MD via an Amazon Web Services (AWS) server located in Ashburn, VA. Panel **b** created in BioRender; Thakor, N. https://biorender.com/7828ikd (2026). The schematic in **e** was created with SolidWorks 2021 (Dassault Systèmes). The world map in **o** was created using the Python package ‘Plotly’. Scale bar, 500 μm (**f**). H, high; L, low.

**Extended Data Fig. 1:**
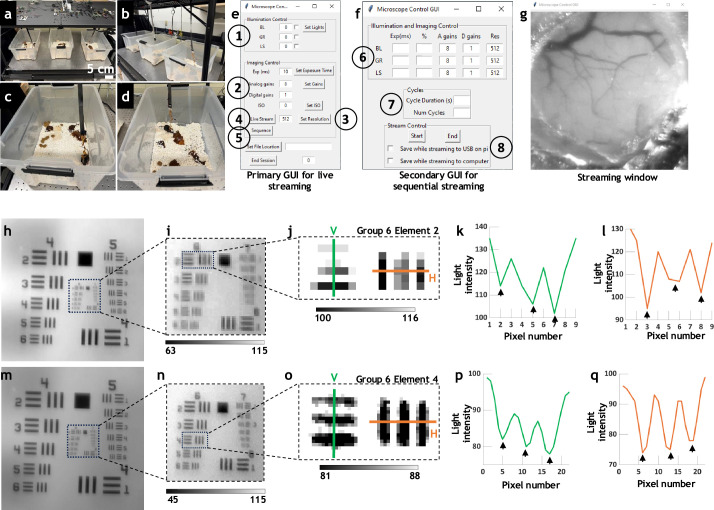
Neurosurveillance via CloudScopes. (**a**) Front and (**b**) side view of a bank of three CloudScopes that are conducting neurosurveillance. (**c**-**d**) Close ups showing freely behaving mice bearing CloudScopes. (**e**) The main GUI window for operating the CloudScope in ‘livestream’ mode. Key components include: (1) the illumination control panel; (2-3) the image sensor control panel; (4) livestream button; and (5) sequential stream button. The illumination control panel consists of boxes for setting the brightness of each illumination source BL, GR, and LS, and check-boxes to select the appropriate source. Once an illumination source and brightness level are selected, the user can click the ‘set lights’ button in (1) to switch on the selected illumination source. The image sensor control panel (2-3) consists of text boxes for specifying the exposure time (ms) and analog and digital gains (1–8, in integer steps) for the image sensor. Once a value is entered into the text box, the user can use the button to its right to set that value. Once these parameters are set, the user can click on the ‘livestream’ button to start image acquisition. This will open a ‘streaming window’ as shown in (**g**), which is continuously updated. In addition, the main window has a ‘sequence’ button, which the user can press to open a secondary window that allows the CloudScope to be operated in the ‘sequential stream’ mode, during which images from multiple channels can be acquired sequentially. (**f**) The sequential stream GUI consists of: (6) an ‘illumination and image sensor control’ panel; (7) a panel to specify the duration and the number of sequential cycles; and (8) options for saving images. (**g**) The streaming window is continuously updated with the latest image acquired with the CloudScope. (**h**) An image of the 1951 USAF positive optical target acquired with the CloudScope at 512 × 512-pixel resolution under green light illumination. (**i**) A magnified view of the optical target showing element 2 in group 6. (**k**-**l**) Intensity profiles along the line ROI V (**k**) and H (**l**), respectively, drawn across group 6’s element 2 corresponding to a resolution of 71.8 line-pairs per mm, or a 6.96 µm ( ~ 7 µm) line width. Arrowheads mark three distinct local minima corresponding to each line segment in the horizontal and vertical elements. (**m**-**q**) Shows a similar analysis for images of the optical target acquired via the CloudScope at 1536 × 1536-pixel resolution, with line ROIs drawn across the target’s group 6, element 4, corresponding to 90.5 line-pairs per mm or a resolvable line width of 5.5 µm.

An IoT architecture (Fig. 1a) permits CloudScopes connected to the internet via Wi-Fi, to be used individually (Fig. 1b) or assembled into a ‘miniscope bank’, for example in an animal facility or a lab (Extended Data Fig. 1a–d and Supplementary Video 1). Users can log into a CloudScope server from anywhere in the world, set image acquisition parameters, initiate image acquisition and observe in vivo experiments in real-time via a graphical user interface (GUI; Extended Data Fig. 1e–g). The CloudScope (Fig. 1c) weighs <3.5 g and can be mounted on the head of a freely behaving mouse (Fig. 1d) to acquire images over a wide field of view (FoV; ~3 × 3 mm^2^), at a high spatial resolution (~5.5–7 µm; Extended Data Fig. 1h–q). Supplementary Table 2 provides a comparison of the CloudScope with extant miniscopes. To ensure affordability and widespread adoption, CloudScope’s control circuitry was implemented using hobbyist electronics and off-the-shelf components. A flexible printed circuit board (PCB) (Fig. 1b,c, ‘flex PCB’) housed the electronics to control a 10-bit image sensor and allowed unimpeded head motion. A surface mount blue LED (wavelength range = ~453 ± 20 nm) and a green (GR) LED (wavelength range = ~530 ± 20 nm), a vertical cavity surface emitting laser diode (LS, center wavelength = 670 nm or 680 nm), an aspheric lens (focal length = 4.6 mm) and a long-pass filter (cutoff wavelength = 510 nm) enabled multicontrast imaging (pupil size = ~1.1 mm and magnification = ~×0.7) with green fluorescence (FL), intrinsic optical signals^4^ (IOS) and laser speckle contrast^5^ (LSC) (Fig. 1e). Unlike in ref. ^6^, additional PCBs for mounting LEDs were not required, as LEDs were directly soldered on to 32-gauge wires (diameter = ~200 µm; Fig. 1c) and any strain on the solder joints relieved by affixing those wires to 3D-printed holes via cyanoacrylate glue. A 3D-printed slider (~2-mm sliding range; Fig. 1e) and lock screw enabled convenient focusing (Supplementary Fig. 1). The 3D-printed head-mount enabled easy attachment of the CloudScope to the mouse’s head (Fig. 1d and Supplementary Fig. 2a–f, working distance of ~2 mm). A meter-long flat flexible cable (FFC; ~200-µm thickness) connected the CloudScope to a control module mounted on the cage (Fig. 1b), which permitted the animal to move freely during 24-h imaging sessions (Supplementary Videos 2 and 3). The control module handled communication to/from the cloud-server (Fig. 1b and Supplementary Fig. 3). Within it, a Raspberry Pi and a Teensyduino were configured in an ‘initiator–responder’ arrangement to manage image acquisition, Wi-Fi-based image transfer, and illumination control. Acquired images were saved to a USB drive (~64 GB to 1 TB) connected to the Raspberry Pi and analyzed offline.

**Extended Data Fig. 2:**
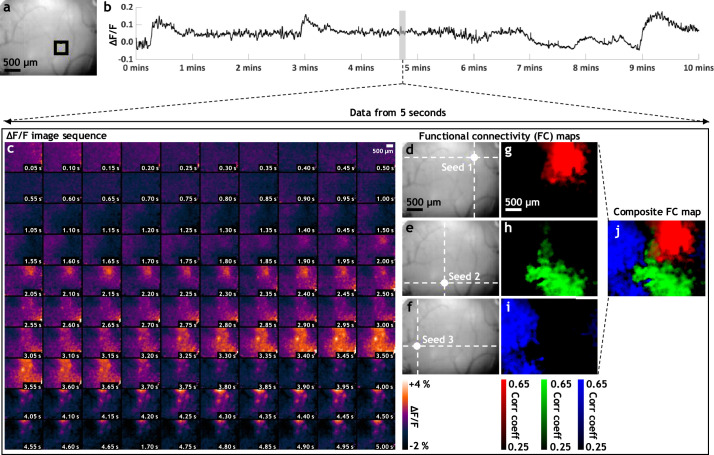
High-speed image acquisition via the CloudScope. CloudScope’s ‘livestream’ mode permitted the acquisition of 128 × 128-pixel resolution (~25 µm/pixel) images of neuronal GCaMP6s fluorescence at 19.03 ± 4.31 frames per second (fps) over >10 minutes. (**a**) Map of mean GCaMP fluorescence computed from the first second of image acquisition. The field-of-view (FoV) was cropped to exclude the skull for visualization purposes. The black box indicates a 15 × 15-pixel ROI. (**b**) 10-minute time-series of changes in GCaMP fluorescence (that is ΔF/F) from the ROI shown in **a**. Data were resampled to a 50 ms resolution for visualization. A 5-second interval from the time-series (indicated by gray shading) was used for subsequent analyses. (**c**) Pseudocolored ΔF/F maps showing changes in neuronal GCaMP fluorescence at 50 ms resolution over 5 seconds. Time stamps for each image are indicated in the lower right corner. (**d**-**f**) Indicate locations of the three seed pixels used to compute maps of microscopic-scale functional connectivity. Functional connectivity maps were generated by computing the Pearson’s correlation coefficient between the ΔF/F time-series for each seed pixel and that for every pixel within the field-of-view (FoV). Prior to computing the correlations, each pixel’s time-series was regressed against the mean time-series of the FoV, and the resulting residual images smoothed with a 5 × 5-pixel mean filter. (**g**-**i**) Functional connectivity maps corresponding to seed pixels in (**d-f**), respectively. Maps are displayed using red, green, and blue pseudocolors. (**j**) A composite of the three functional connectivity maps. Data shown are from animal M4.

The head-mounted CloudScope (Fig. 1d) permitted assessment of multiple neurophysiologic variables, including (1) changes in Neu_ACT_ via GCaMP6s-bound Ca^2+^ in neuropil and neuronal soma^7^ (Fig. 1f and Supplementary Fig. 4) or tracking of fluorescent dyes and cells via the FL channel; (2) mapping changes in CBV (∆CBV, with total hemoglobin content or ∆HbT; Fig. 1g), oxy-/deoxy-hemoglobin content (∆HbO and ∆Hb) and intravascular oxygen saturation (Hb_SAT_) via the IOS channels; (3i) and quantifying changes in CBF (∆CBF) via the LSC channel (Fig. 1h). Users could operate the CloudScope in ‘livestream’ mode to fine tune image acquisition parameters for each contrast mechanism (Supplementary Table 3) or conduct high-speed (up to ~19 fps; Extended Data Fig. 2) imaging using a single contrast mechanism. Examples include discriminating cerebral arterioles from venules by tracking the transit of an intravenously injected FL tracer (size 512 × 512 pixels, resolution = 7 µm, speed = ~9 fps; Supplementary Video 4) or visualizing microscopic-scale functional connectivity patterns with high temporal resolution image acquisitions (size 128 × 128 pixels, resolution = ~25 µm, speed = ~19 fps; Extended Data Fig. 2 and Supplementary Video 5). Users could then switch to a ‘sequential stream’ mode to automatically cycle through each channel for a predefined period (for example every ~5 s) while minimizing overheating of the image sensor (Supplementary Fig. 5). Cyclically acquired images were resampled at fixed time steps to correct for minor variations in frame rate and obtain concurrent multicontrast datasets. CloudScope operation (for example for >10 min of live streaming or >24 h of sequential streaming; Supplementary Videos 6 and 7) did not result in excessive heating or photobleaching (Supplementary Figs. 6 and 7).

**Extended Data Fig. 3:**
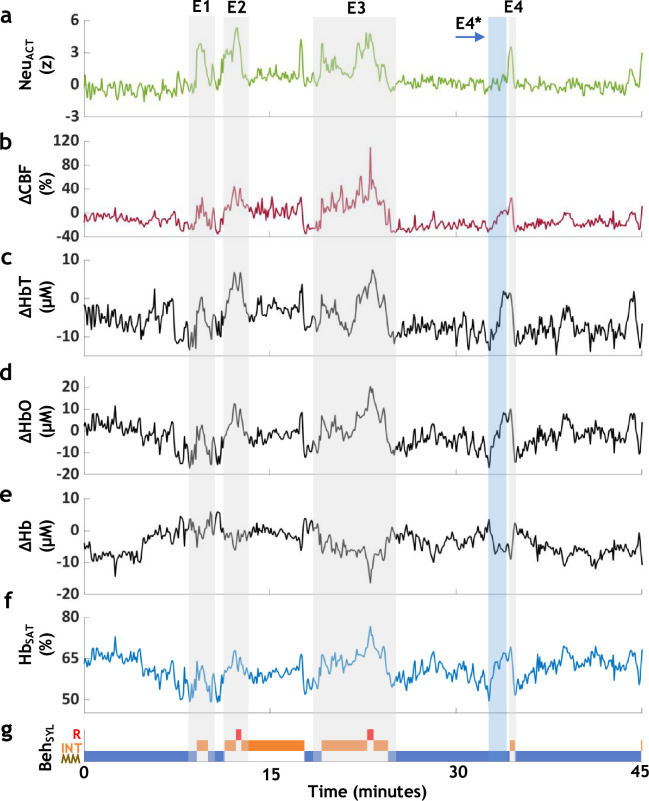
Relating animal behavior to changes in neuronal activity and hemodynamics. A representative 45-min time-series of: (**a**) Neu_**ACT**_, (**b**) CBF, (**c**) ΔHbT, (**d**) ΔHbO, (**e**) ΔHb, and (**f**) Hb_SAT_ from a freely behaving animal (M2). Data are averages over parenchymal regions (that is regions that excluded visible blood vessels). (**g**) Time-series of the corresponding behavioral syllables (Beh_SYL_), annotated as being either minimally mobile (MM), running (R) or intermediary (INT). Periods of MM, INT, and R were color-coded in blue, orange, and red, respectively. Five epochs, E1, E2, E3, E4, and E4* are also indicated.

**Extended Data Fig. 4:**
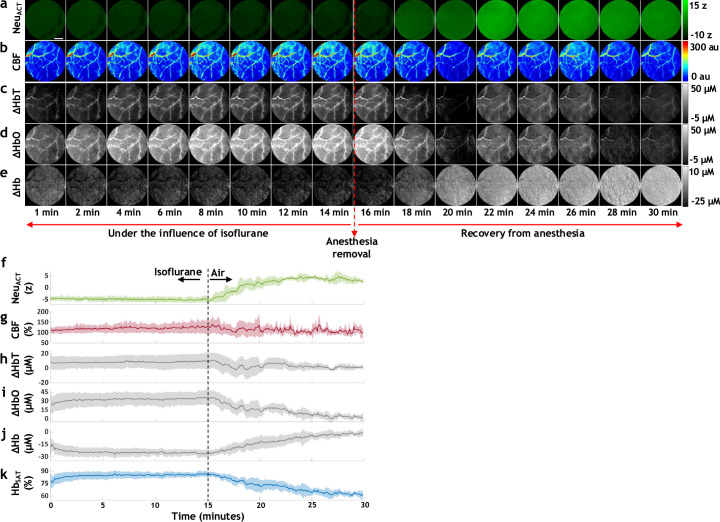
Interrogating changes in brain function during recovery from anesthesia. Maps of: (**a**) Neu_ACT_, (**b**) CBF, (**c**) ΔHbT (that is ΔCBV), (**d**) ΔHbO, (**e**) ΔHb in a representative animal (M2) during recovery from isoflurane anesthesia. Anesthesia was maintained via inhalation of ~1.5% isoflurane at ~1 L/min. Mean time-series of: (**f**) Neu_ACT_, (**g**) CBF, (**h**) ΔHbT, (**i**) ΔHbO, (**j**) ΔHb, and (**k**) Hb_SAT_ computed for a cohort of n = 5 animals (M1-M5). Data are means over parenchymal regions (that is excluding visible blood vessels). Thick lines and shaded areas show mean ± standard deviation over n = 5 animals (M1-M5). For visualization purposes, noise in CBF maps was suppressed using Eq. (20) and that in Hb_SAT_ images was suppressed by applying a nonlocal means filter (neighborhood size=300 × 300 μm, respectively. Scale bar=500 μm.

Animal activity was captured via a separate ‘behavioral camera’ (Fig. 1i), and annotated offline into three ‘behavioral syllables’^8^ (Behavior_SYL_): minimally mobile (MM), intermediate (INT) or running (R) (Fig. 1n). These technical advances permitted us to continuously correlate Neu_ACT_, CBV, CBF and Hb_SAT_ changes with animal behavior over >24 h (Fig. 1j–n). As expected^9^, epochs of increased animal activity were accompanied by elevated Neu_ACT_ and CBF, vasodilation (increased CBV), increased HbO, decreased Hb, and increased Hb_SAT_ (for example epochs E1, E2, E3 and E4; Extended Data Fig. 3). In some instances, elevated CBF, CBV and Hb_SAT_, which may be linked to the hemodynamic correlates of sleep or arousal^10^, were observed without proportionate changes in Neu_ACT_ or behavior (for example epoch E4*; Extended Data Fig. 3). Conversely, we observed a suppression of Neu_ACT_ concurrent with an increase in Hb_SAT_ under isoflurane anesthesia (in *n* = 5 mice) that was consistent with prior reports of decreased electroencephalogram (EEG) activity and reduced cerebral metabolic rate of oxygen induced by that anesthetic^11^ (Extended Data Fig. 4). Collectively, long-term neurosurveillance via multicontrast imaging as well as the CloudScope’s remote access capabilities (Fig. 1o) create a platform for interrogating the complex interplay between Neu_ACT_ and vascular dynamics in freely behaving animals.

### Deep learning can predict individual animal behavior from 24-h neurosurveillance data

**Fig. 2:**
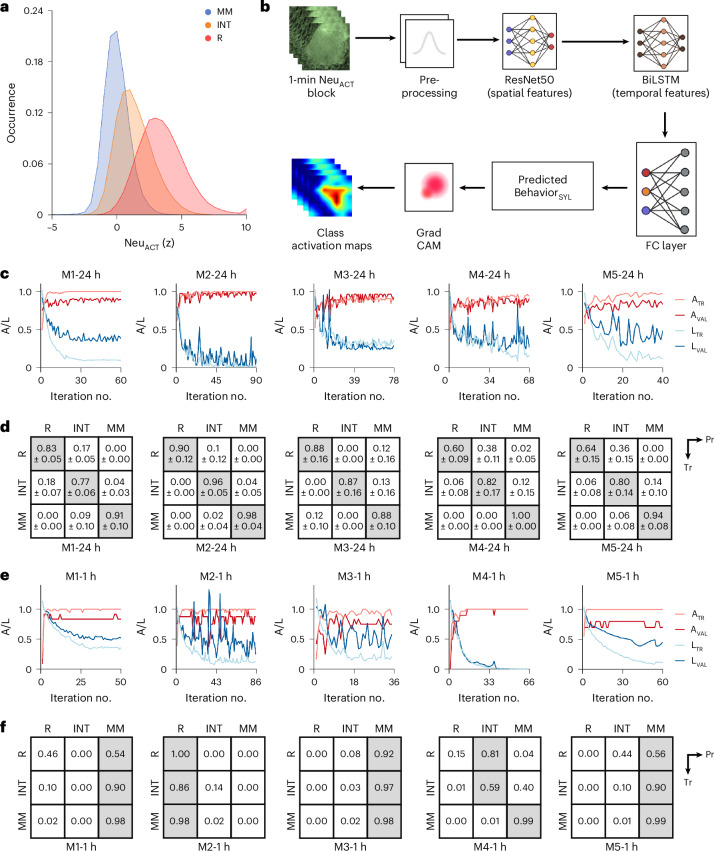
Deep learning can predict individual animal behavior from 24-h neurosurveillance data. **a**, Distributions of Neu_ACT_ (μ Neu_ACT_) corresponding to MM, INT and R Behavior_SYL_ over 24 h. Data shown are from M2. **b**, Schematic illustrating the DL pipeline. First, 1-min intervals, during which, an animal consistently maintained a Behavior_SYL_ (MM, INT or R) were identified and the corresponding Neu_ACT_ images grouped together to create 1-min ‘Neu_ACT_ blocks’. Key components of the pipeline such as the pre-processing steps, the ResNet50 unit, the BiLSTM unit and the FC layer are also shown. Additionally, the class scores created at the end of the DL pipeline were input into a gradient class activation mapping (Grad-CAM) unit to identify the spatial location of features that the DL pipeline utilized from the Neu_ACT_ time-series to predict each Behavior_SYL_. **c**, Training and validation accuracy and loss curves (A_TR_, T_VAL_, L_TR_ and L_VAL_, respectively) generated from each animal M1–M5 (*n* = 5) for a representative fold. **d**, Corresponding confusion matrices showing the accuracies achieved for each Behavior_SYL_ or class (Tr, true; Pr, predicted). Confusion matrices show mean ± s.d. over the 5× folds. **e**,**f**, Analogous accuracy and loss curves for training and validation (**e**) and confusion matrices (**f**) when the same DL pipeline was trained using only the first hour of Neu_ACT_ data. H, high; L, low. The highest accuracy in each row of the confusion matrices is shaded in gray. Components of panel **b** created in BioRender. Thakor, N. https://biorender.com/j8n8zv9 (2026).

DL is fast gaining popularity as an approach for predicting animal behavior from neuroimaging data^12^. While non-DL methods such as complex-PCA^13^, dynamic functional connectivity (dFC)^14^ or co-activation patterns (CAPs)^15^, can be used for behavioral classification, tradeoffs between their specificity versus sensitivity, or the need to identify a reference region often precludes their use for predicting animal behaviors (for example MM, INT or R) from short-duration (for example 1 min) and limited FoV (only 3 × 3 mm^2^) neurosurveillance data. Conversely, the limited amount of neuroimaging data available per animal from benchtop-based neuroimaging systems (for example <2 h) often necessitates pooling data from multiple animals^12^, making it unfeasible to account for characteristics unique to individual animals. To address this, we hypothesized that we could use neurosurveillance-based data for optimizing a DL model pretrained on nonbiological inputs to predict ‘individual animal’ behavior from its own neuroimages (Fig. 2a and Supplementary Fig. 8a–e).

**Extended Data Fig. 5:**
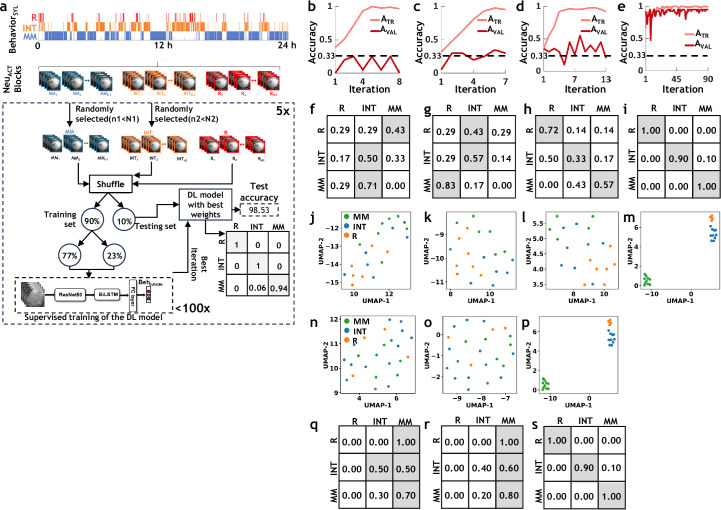
Training a deep learning (DL) model on CloudScope-acquired data, evaluating its performance, and validating via sanity checks. (**a**) First, Neu_ACT_ images corresponding to 1-minute intervals during which the animal-maintained a given behavioral syllable (that is Beh_Syllable_) were grouped to create ‘Neu_ACT_ blocks’, that is MM_1_… MM_N1_, INT_1_… INT_N2_, and R_1_… R_N3_, for each Beh_Syllable_ MM, INT, and R, respectively. Typically, N1 > N2 » N3 over a 24-h period, which resulted an imbalanced dataset. Therefore, to circumvent the deleterious effects of this imbalance, we employed a 5-fold validation process, during which a subset of the dataset comprising of similar numbers of Neu_ACT_ blocks corresponding to each Beh_Syllable_ (that is n1 ~ n2 ~ N3) was randomly selected 5 times, and for each such selection, the DL pipeline was trained, and its performance evaluated. Specifically, each randomly selected dataset was shuffled, and partitioned into training (90%) and testing (10%) datasets. Next, over a maximum of 100× iterations the training dataset was further subdivided into training and validation datasets (Supplementary Table 5). The training dataset was input into a DL-pipeline that included a ResNet50, a BiLSTM, and a fully connected (FC) layer for behavioral prediction, and the DL model’s performance evaluated on the validation dataset, while its errors were back-propagated through a loss function. Model weights that provided the highest validation accuracy were then selected, and the performance of the DL pipeline with these weights assessed on the test dataset to obtain ‘test’ accuracy and a confusion matrix across the three behavioral syllables. Pr=predicted Behavior_SYL_, Tr=true Behavior_SYL_. (**b-m**) Sanity check #1-Training the DL models on shuffled labels. (**b-d**) Training and validation accuracies (that is A_TR_ and A_VAL_, respectively) for three independent training runs of the ResNet50-BiLSTM model with 40 Neu_ACT_ blocks for which behavioral labels were randomly shuffled. The chance level (that is 0.33) is depicted by a dashed, black line. (**e**) Similar A_TR_ and A_VAL_ curves for the same model trained on unshuffled data. (**f-h**) Confusion matrices for the three DL models trained on shuffled data, and (**i**) confusion matrix for the DL model trained on unshuffled data. (**j-l**) UMAPs for the DL models trained on shuffled data. (**m**) UMAP for the DL model trained on unshuffled data. (**n-s**) Sanity check #2-Testing model performance after shuffling all frames. (**n-o**) UMAPs when the ResNet50-BiLSTM model was tested on the randomly shuffled 24-h Neu_ACT_ images. Results are shown for two randomization runs. (**p**) UMAP when the same DL model was tested on the unshuffled neurosurveillance dataset. (**q-r**) Corresponding confusion matrices showing the model’s performance for the randomly shuffled data in **n-o**. (**s**) Confusion matrix showing the model’s performance for the unshuffled neurosurveillance dataset. The entry with the highest accuracy in each row of the confusion matrices is shaded in gray. Both sanity checks were implemented using data from mouse M2.

**Extended Data Fig. 6:**
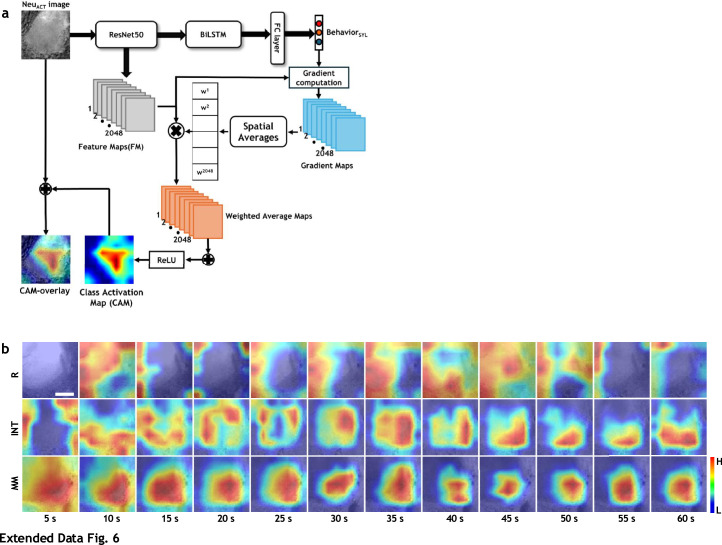
Interpreting DL-based behavioral predictions via the Grad-CAM algorithm. (**a**) First, each Neu_ACT_ block was passed through the trained DL pipeline. Next, for each image in the Neu_ACT_ block, feature maps (FMs) produced by the ResNet50 module were extracted (n = 2048 maps, 7 × 7 pixels per map), and the gradient of the behavioral class scores computed with respect to these FMs were. The resulting ‘gradient maps’ (n = 2048) were then spatially averaged to create weighing factors w^1^… w^2048^, and each FM multiplied by its corresponding weight to create weighted average maps. These maps were then summed and passed through a rectified linear unit (ReLU) to generate the class activation map (CAM), which was overlaid on the original image (that is CAM-overlay) to visualize areas within the Neu_ACT_ image that were most relevant to the behavior prediction. (**b**) Representative sequences of class activation maps (CAMs) for Neu_**ACT**_ blocks corresponding to behavioral syllables MM, INT, and R. Scale bar=1 mm. H=high, L=low.

We utilized a pipeline consisting of two DL modules: a ResNet50 (ref. ^16^) (for extracting spatial features) and a BiLSTM^17^ (for extracting temporal features) (Fig. 2b, Extended Data Fig. 5a and Supplementary Table 4). First, for each animal Neu_ACT_ images acquired from the murine motor and somatosensory cortices (3 × 3 mm^2^ FoV centered −2/+2 mm anteroposterior (AP)/mediolateral (ML) with respect to the bregma) were grouped into continuous 1-min periods during which the animal exhibited a single Behavior_SYL_ (MM, INT or R). These 1-min image sequences (‘Neu_ACT_ blocks’), were randomly split into training (90%) and testing (10%) subsets, and the training dataset input into the DL pipeline. A fully connected (FC) layer at the end of the ResNet50/BiLSTM modules predicted the Behavior_SYL_ attributable to each Neu_ACT_ block (Fig. 2b). We also implemented a DL interpretation technique known as gradient-weighted class activation mapping^18^ (Grad-CAM), which identified regions within each image of the Neu_ACT_ block that contributed to the classification of the associated Behavior_SYL_ (Extended Data Fig. 6). The pipeline was trained, validated and tested across 5× folds per animal (Fig. 2c shows the accuracy and loss curves from training/validation phases during a representative fold).

We were able to repeatably achieve high behavioral prediction accuracies in *n* = 5 individual animals (the average per-class accuracy (mean ± s.d.), computed across five-folds was 84 ± 4%, 94 ± 4%, 88 ± 13%, 81 ± 5% and 79 ± 3%, for mice M1–M5 respectively, evaluated 5× on a random selection of 10% of each animal’s data unseen by the DL pipeline). Corresponding macro-averaged F1 scores across five-folds are summarized in Supplementary Table 6. As shown by the confusion matrices (Fig. 2d), the prediction accuracies achieved with 24 h of Neu_ACT_ data were high across all Behavior_SYL_ for each animal. We show that in contrast, prediction accuracies deteriorated for the same DL model when it was trained on Neu_ACT_ blocks with randomized behavioral labels (Extended Data Fig. 5b–m). These performance indices demonstrate that training with neurosurveillance data was essential to enable accurate behavioral predictions. Similarly, retaining the temporal continuity in neurosurveillance data was necessary to ensure accurate DL-based predictions (Extended Data Fig. 5n–s). Training the same DL pipeline with only the first hour of Neu_ACT_ data (without 24-h neurosurveillance), resulted in prediction bias and substantially lower prediction accuracies for each animal (Fig. 2e,f, average per-class accuracies of 48%, 38%, 34%, 58% and 36%, respectively for mice M1–M5 that were evaluated using the remaining 23 h of each animal’s data unseen by the DL pipeline). Collectively, these results demonstrate the feasibility of harnessing a ‘precision approach’^19^, wherein neuroscientists can ‘individualize’ DL-based behavioral predictions when characterizing the neurological correlates of natural behavior.

### Hemodynamic changes and neuronal activity did not always colocalize during natural behavior

Neuroimaging in anesthetized or head-fixed animals has shown that ultraslow fluctuations (≤0.1 Hz) in Neu_ACT_ lead to vasomotion of nearby arterioles to create synchronized changes in CBV spanning hundreds of microns^20^. Analogous Neu_ACT_-coupled hemodynamic changes (functional hyperemia) constitute the basis of many widely used neuroimaging methods, including blood oxygen-level dependent (BOLD) functional magnetic resonance imaging (fMRI)^21^ and IOS imaging^4^. While ‘twitches, blinks and fidgets’^22^ or shifts in an animal’s sleep state^10^ have been shown to alter brain hemodynamics in head-fixed mice, the impact of animal behavior on neurovascular coupling in the ultraslow frequency range remains poorly characterized. To address this, we conducted the neurosurveillance of Neu_ACT_ and vascular changes that occur in the ultraslow frequency range within the murine motor and somatosensory cortices (3 × 3 mm^2^ FoV centered −2/+2 mm AP/ML with respect to the bregma, *n* = 5 mice, denoted by M1–M5) of freely behaving animals. Unlike conventional studies in awake animals that were often limited to 10–15 min of continuous imaging^2^, neurosurveillance enabled neurovascular imaging over a ~ 100× longer duration (1,440 min or 24 h) in the same animal (see time-series in Fig. 1j–m and Supplementary Video 6 for data from a representative animal). All data were analyzed within 15-min epochs, so that each epoch was comparable to a conventional 15-min miniscope-based imaging experiment. The fraction of time the animal was active in each epoch (F_ACTIVE_, when the animal was either in an INT or R state) was used as a metric of the animal’s behavior.

**Fig. 3:**
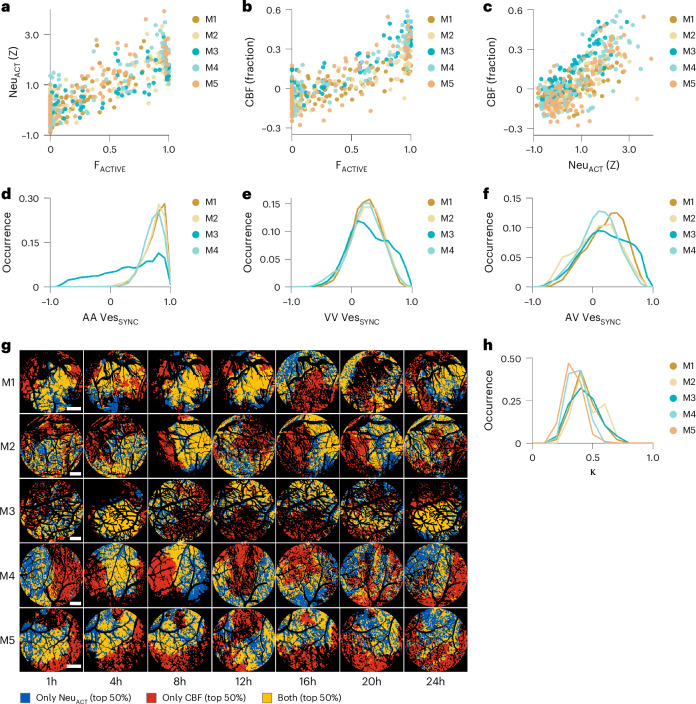
Hemodynamic changes and neuronal activity did not always colocalize during natural behavior. **a**–**c**, Scatter-plots of F_ACTIVE_ versus Neu_ACT_ (**a**); F_ACTIVE_ versus CBF (**b**); and Neu_ACT_ versus CBF (**c**), over 24-h of natural behavior for *n* = 5 animals, M1–M5. Data points for Neu_ACT_ and CBF are averages computed over 15-min epochs from the cortical parenchyma (that is, the FoV excluding surface vessels). Each 15-min epoch approximated a conventional ‘short-duration’ miniscope imaging experiment. F_ACTIVE_ was the fraction of time the animal was in a non-MM state (either INT or R) during each epoch and reflects the animal’s activity level. Distributions of artery–artery (AA; **d**), vein–vein (VV; **e**) and artery–vein (AV; **f**) vasomotor synchronization (Ves_SYNC_) over 24 h for *n* = 4 mice, M1–M4, for which we could discriminate arteries from veins. Ves_SYNC_ between vessel pairs was defined as the Pearson’s correlation coefficient between the time-series of their diameter changes during each epoch. **g**, Timelapse images of the overlap (yellow) between cortical areas that exhibited peak Neu_ACT_ (top 50%, blue) and CBF (top 50%, red) in *n* = 5 mice over 24 h. **h**, Distribution of the overlap coefficient (*κ*) for each animal. Note, overlap coefficients were computed on 64 × 64-pixel images. Scale bar, 500 μm (**g**).

At the macroscopic spatial scale (over the entire 3 × 3 mm^2^ FoV), Neu_ACT_ and CBF were strongly correlated with their F_ACTIVE_ levels in each animal (Fig. 3a,b, Mean Spearman’s correlation coefficient ± s.d. = 0.83 ± 0.03 and 0.77 ± 0.07 for Neu_ACT_ versus and CBF versus F_ACTIVE_, respectively, *n* = 5 animals). At this spatial scale, Neu_ACT_ and CBF levels were also strongly correlated in each animal (Fig. 3c; mean Pearson’s correlation coefficient ± s.d. = 0.79 ± 0.05, *n* = 5 mice). Similar to previous observations^23^, the positive Pearson’s correlation coefficient suggests that in each mouse, changes in average Neu_ACT_ and CBF occurred synchronously.

To determine whether these CBF changes were attributable to the vasomotion of surface arteries or veins, we developed a technique to quantify arterial/venular diameter changes from widefield images of total hemoglobin content acquired with the CloudScope (Supplementary Fig. 9). This technique enabled us to track in vivo vasomotion, over a FoV (3 × 3 mm^2^) that was 30× larger than that reported in two-photon imaging-based studies^20^ (0.5 × 0.5 mm^2^). Analogous to vasomotor coherence computed in ref. ^20^, we computed the ‘vasomotor synchronization’ (Ves_SYNC_) between pairs of surface vessels in terms of the correlation between their diameter changes during 15-min epochs. Unlike in head-fixed in vivo experiments, wherein diameter changes of cerebral arteries and veins were reported to be coupled^20^, we discovered that Ves_SYNC_ varied substantially in freely behaving animals (Fig. 3d–f; in *n* = 4 animals, M1–M4). Furthermore, the Ves_SYNC_ distribution between arteries versus veins ranged over positive and negative values (Fig. 3f), which was consistent with previous reports of these two vessel categories exhibiting distinct temporal fluctuations and frequency spectra^24^.

Next, we hypothesized that the heterogenous arterial Ves_SYNC_ (for example Fig. 3d) may be associated with spatially heterogeneous Neu_ACT_-induced CBF responses. To test this, we computed the spatial overlap coefficient (*κ*) between parenchymal regions exhibiting peak Neu_ACT_ (top 50%) and peak CBF (top 50%) (Fig. 3g and Supplementary Video 8). This spatial overlap (*κ*) was low, that is 0.40 ± 0.04 for all (*n* = 5) animals (Fig. 3h), which was suggestive of poor colocalization between peak CBF and Neu_ACT_ in freely behaving animals. Such low *κ* values were consistent with prior reports describing the existence of non-neuronal origin hemodynamic fluctuations^25^ and the spatial ‘overspill’ characteristic of the functional hyperemic response^23^. Collectively, these findings demonstrate that while Neu_ACT_ and CBF remain coupled in the time domain at the macroscopic scale, they can exhibit low spatial overlap at the microvascular scale during natural animal behavior.

### Neurosurveillance reveals the trajectory of seizure-induced neurovascular disruption and recovery

**Fig. 4:**
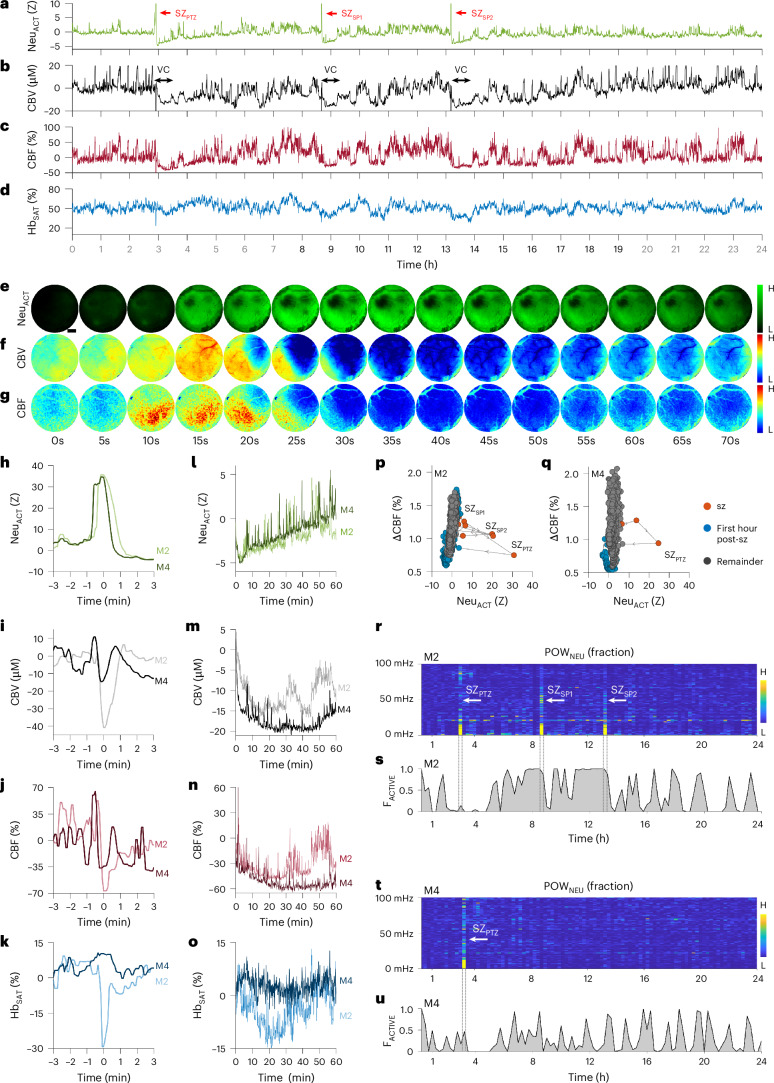
Neurosurveillance reveals the trajectory of seizure-induced neurovascular disruption and recovery. **a**–**d**, Representative 24-h time-series illustrating changes in Neu_ACT_ (**a**), CBV (**b**), CBF (**c**) and Hb_SAT_ (**d**) in a murine seizure model. Two spontaneous seizures (SZ_SP1_ and SZ_SP2_) were observed at ~5.5 h and ~10.5 h after recovery from an initial seizure (SZ_PTZ_) induced via an i.p. injection of PTZ, a GABA_A_ antagonist. Induced and spontaneous seizures were associated with vasoconstriction (‘VC’). Mean time-series were computed for the cortical parenchyma (over the FoV excluding surface vessels) and smoothed with a 30-s median filter for visualization. **e**–**g**, Images illustrating the spatiotemporal evolution of Neu_ACT_, CBV and CBF during a PTZ-induced seizure, wherein brief periods of vasoconstriction (**f**) and hypoperfusion (**g**) accompanied the rise and fall of Neu_ACT_ during seizure progression (**e**). **h**–**o**, Time-series illustrating changes in Neu_ACT_, CBV, CBF and Hb_SAT_, during a primary seizure (**h**–**k**) and 1 h after seizure cessation (**l**–**o**), respectively. Data are shown for two animals, M2 and M4. Time-series in **h**–**k** were resampled at 5 s and smoothed with a 15-s median filter for visualization. Time-series in **l**–**o** were resampled at 1 min. **p**,**q**, The 24-h trajectories of the ‘neurovascular landscape’ of the two mice M2 (**p**) and M4 (**q**) were visualized on a Neu_ACT_ versus CBF ‘state-space’. On this state-space we plotted seizures (orange dots), their 1-h aftermath (blue dots), and instances of nonseizure or healthy neurovascular activity (gray dots). These state-space trajectories illustrate the occurrence of two spontaneous seizures (SZ_SP1_ and SZ_SP2_) in animal M1 following the primary PTZ-induced seizure (SZ_PTZ_), in contrast to mouse M2, which did not exhibit any spontaneous seizures. The points in the state-spaces were the mean Neu_ACT_ and CBF computed over 2 min for visualization purposes. **r**–**u**, The power spectra of Neu_ACT_ (**r**,**t**) and the corresponding changes in behavioral activity (F_ACTIVE_) (**s**,**u**) for each animal M2 and M4, respectively. Scale bar, 500 μm (**e**). H, high; L, low.

**Extended Data Fig. 7:**
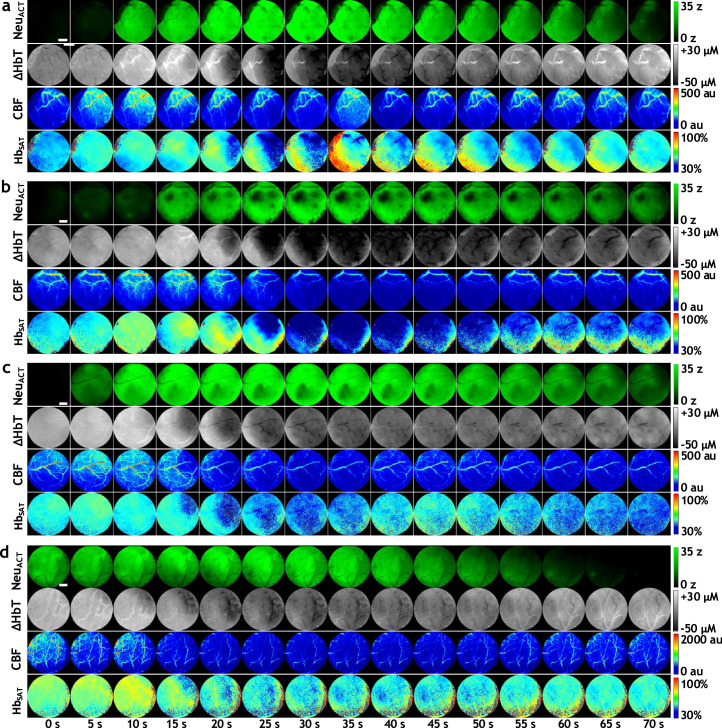
Seizure-induced neurovascular disruptions. (**a**-**d**) Panels showing Neu_ACT_, ΔHbT (that is CBV), CBF, and Hb_SAT_ time-series during PTZ-induced seizures in animals M1-M4, respectively. For visualization purposes, noise in CBF maps was suppressed using Eq. (14) and that in Hb_SAT_ images was suppressed by applying a nonlocal means filter (neighborhood size = 300 × 300 μm). Scale bar=500 μm.

**Extended Data Fig. 8:**
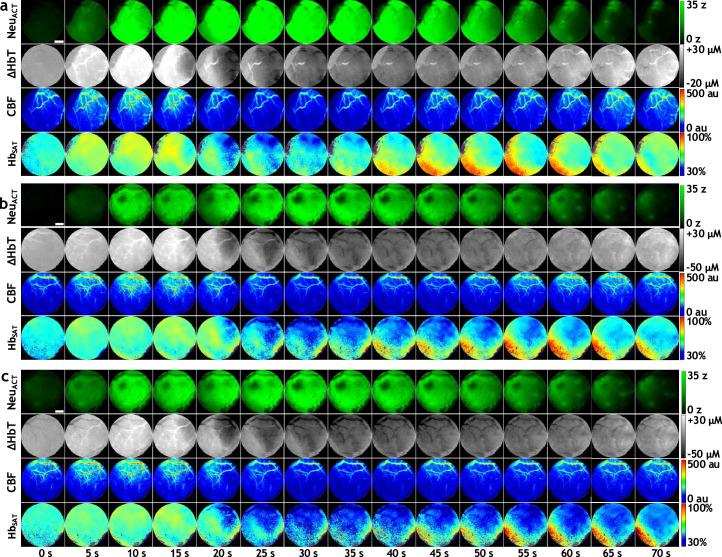
Neurovascular changes during spontaneous seizures. (**a**-**c**) Panels showing Neu_ACT_, ΔHbT (that is ΔCBV), CBF, and Hb_SAT_ time-series during three spontaneous seizures: one in animal M1 (**a**), and two in animal M2 (**b**-**c**). For visualization purposes, noise in CBF maps was suppressed using Eq. (20) and that in Hb_SAT_ images was suppressed by applying a nonlocal means filter (neighborhood size=300 × 300 μm). Scale bar=500 μm.

Recent evidence suggests that seizures, which are primarily thought of as neuroelectric events, result in substantially dysregulated cerebral hemodynamics^26^. Yet, our understanding of a seizure’s vascular impact remains incomplete because most seizure experiments study brain function only for a few minutes (from seizure initiation to cessation)^27^. In contrast, neurosurveillance enabled us to continuously interrogate the brain, before, during and after the induction of a seizure (SZ_D_, via an intraperitoneal (i.p.) injection of the drug pentylenetetrazol (PTZ), a GABA_A_ antagonist), over an entire day (Fig. 4a–d, from mouse M2). Using CloudScope’s multicontrast capabilities, we observed extensive in vivo changes in Neu_ACT_ with concomitant changes in CBV, CBF and Hb_SAT_, as well as the occurrence of multiple spontaneous seizures (for example, SZ_P1_ and SZ_P2_) that would have been missed without 24-h neurosurveillance. We visualized the spatiotemporal evolution of neurovascular changes during SZ_D_ (Fig. 4e–g, ~75 s), wherein one could identify distinct periods of vasoconstriction (Fig. 4f, 20–40 s) and hypoperfusion (Fig. 4g, 20–40 s) coincident with seizure onset and culmination (Fig. 4e, 0–45 s), as well as the partial recovery of these hemodynamic changes (Fig. 4f,g, 50–70 s) after seizure termination (Fig. 4e, 50–70 s). Similar changes in Neu_ACT_, CBV, CBF and Hb_SAT_ were observed for four SZ_D_ and three SZ_SP_ in a cohort of *n* = 4 animals (M1–M4) (Extended Data Figs. 7 and 8 and Supplementary Videos 9 and 10). We also developed a neurosurveillance dashboard to concurrently visualize changes in these neurophysiological variables (Supplementary Video 11). The recovery of each neurophysiological variable to its pre-seizure baseline from the drug-induced (SZ_D_), and spontaneous (SZ_SP1_ and SZ_SP2_) seizures took approximately 1 h.

**Extended Data Fig. 9:**
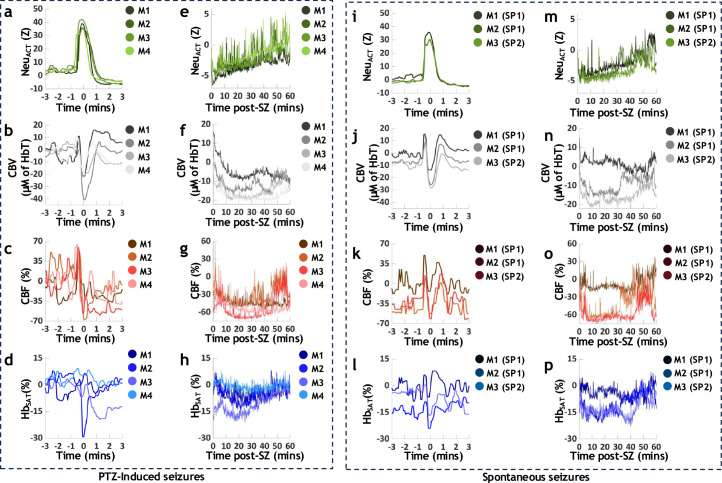
Trajectories of neurovascular disruption in drug-induced vs. spontaneous seizures, and their recovery. Time-series of: (**a**) Neu_ACT_, (**b**) ΔHbT (that is ΔCBV), (**c**) CBF, and (**d**) Hb_SAT_, during PTZ-induced seizure in animals M1-M4. Time-series show spatial averages computed over all parenchymal pixels. (**e**-**h**) Similar graphs showing the recovery of neurovascular function during the first hour after seizure cessation. Time-series of (**i**) Neu_ACT_, (**j**) ΔHbT (that is ΔCBV), (**k**) CBF, and (**l**) Hb_SAT_, during n = 3 spontaneous seizures observed in animals M1 (n = 1) and M2 (n = 2), after the cessation of the drug-induced seizure. (**m-p**) Similar graphs showing the recovery of neurovascular function during the first hour after seizure cessation. Data from different animals are aligned by assigning the time of peak Neu_ACT_ during each seizure to t = 0.

Substantially different degrees of hemodynamic change (Fig. 4i–k, for example in mice M2 and M4) occurred during seizures that showed similar Neu_ACT_ dynamics (Fig. 4h). For example, mouse M2 (seizure score = 5 on a revised Racine scale^28^) exhibited greater vasoconstriction (Fig. 4i) and decreased Hb_SAT_ (Fig. 4k) relative to those of M4 (seizure score = 5), despite both mice exhibiting similar seizure-induced neuronal activations (Fig. 4i). The overall CBF changes between M2 and M4 were also different (Fig. 4j). Imaging revealed neurovascular changes between all drug-induced and spontaneous seizures (Extended Data Fig. 9a–d,i–l). Moreover, consistent with previous studies demonstrating generalized EEG suppression^29^ and cortical arteriolar vasoconstriction^26^, the first ~60 min following seizure termination were characterized by depressed Neu_ACT_ (Fig. 4l), sustained vasoconstriction (Fig. 4m) and reduced CBF (Fig. 4n). These alterations translated into a reduction in Hb_SAT_ for M2, but not for M4 (Fig. 4o). A similar trend in Neu_ACT_, CBV and CBF was observed for all seizures (Extended Data Fig. 9e–h,m–p). Taken together, these data suggest that the conventional practice of using Neu_ACT_-centric techniques for studying seizures (for example EEG) may result in an incomplete characterization of the underlying neuropathology.

To highlight this drawback, we mapped the neurovascular trajectory of each mouse via a Neu_ACT_ versus CBF state-space (Fig. 4p–q). Gray, orange and blue markers identifying healthy, seizure and post-seizure depression phases show the emergence of multiple spontaneous seizures in M2, but not M4. Power spectrograms of Neu_ACT_ (Fig. 4r,t) illustrate that in the current experimental model, spontaneous seizures were characterized by elevations in low-frequency (0–15 mHz) Neu_ACT_ like those observed during the drug-induced seizures and could also be accompanied by diminished activity in their aftermath (Fig. 4s,u). Collectively, these observations underscore the utility of neurosurveillance as a tool for holistically characterizing the neurovascular trajectories and heterogeneity of CNS neuropathologies such as seizures.

### Neurosurveillance of brain tumor evolution

**Fig. 5:**
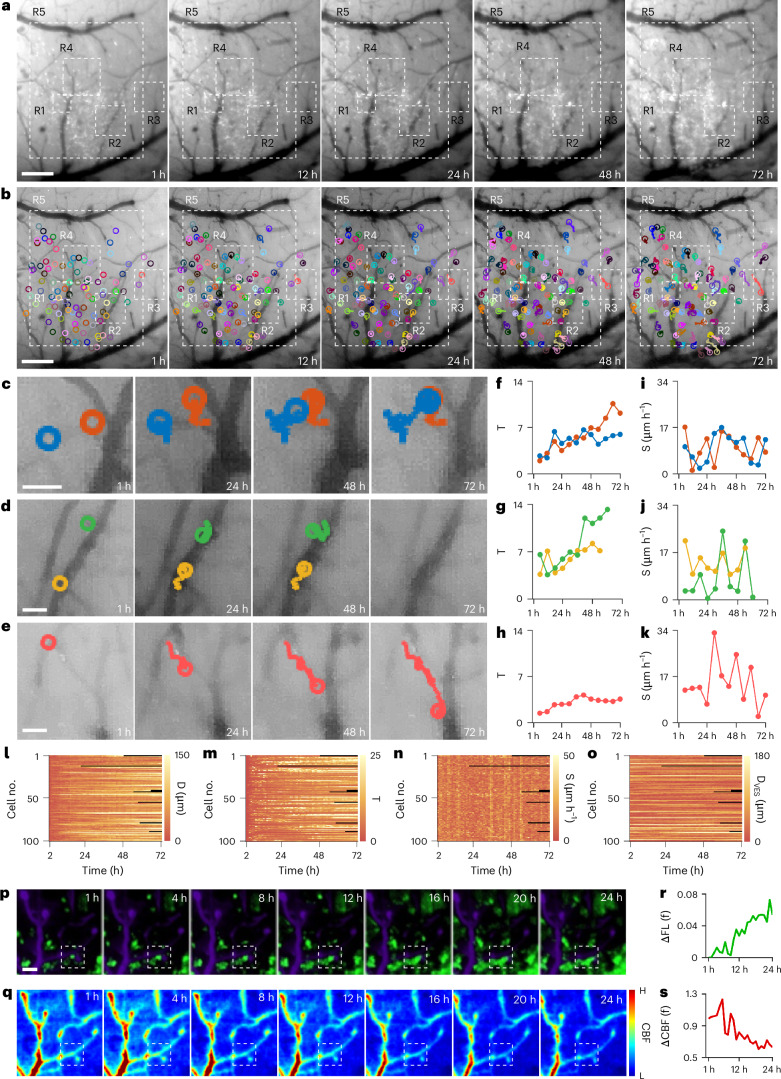
Neurosurveillance of brain tumor evolution. **a**, Timelapse images of glioma cell migration and proliferation in a brain tumor-bearing animal acquired over 72 h via neurosurveillance during the TI phase. Images are shown in a gray scale, with bright spots indicating fluorescent GL261–GFP murine glioma cells and hatched rectangles indicating regions of interest (ROIs R1–R5) within the brain tumor microenvironment (BTME) for subsequent analyses. **b**, Pseudocolored in vivo migration trajectories of 100 GL261–GFP cells within this BTME that were tracked over 72 h. For visualization purposes, glioma cell trajectories were overlayed on a map of the cerebral microvasculature. **c**–**e**, The CloudScope’s high spatial resolution (~7 μm) enabled us to identify unique cellular-scale events, such as glioma cells merging (**c**, cells C1 and C2, in R1), glioma cells intravasating into pre-existing blood vessels (**d**, cells C3 and C4 in R2) and glioma cells migrating over long distances (>100 μm) to other brain regions (**e**, cell C5 in R3). **f**–**h**, Tortuosity (T) versus time plots showing the evolution of glioma cell migration over 72 h, for the cells shown in **c**–**e**, respectively. **i**–**k**, Migration speed (S) versus time plot for the same cells. **l**–**o**, In vivo ‘functional assays’ of glioma cell migration during the TI phase, showing ensemble-wide profiles of tumor cell displacement (**l**, ‘D’), tortuosity (**m**, ‘T’), migration speed (**n**, ‘S’) and proximity to blood vessels (**o**, ‘D_VES_’), as a function of time. Timelapse images over a representative 24-h period from ROI R4 during which glioma cells (**p**, green) co-opted and proliferated around pre-existing blood vessels (**p**, purple) that also exhibited reduced CBF (**r**). White hatched lines indicate a ROI for which changes in FL intensity and CBF are plotted in (**q**) and (**r**), respectively. Tumor cell proliferation (**q**, via GFP FL of tumor cells) and CBF reduction (**r**) were evident from these plots. For visualization purposes, pseudocolored images in **p** were generated by sharpening IOS and FL images with a 3 × 3-pixel kernel and combining the inverted IOS image in the red channel with the FL image in the green channel and adjusting their relative intensities. Similarly, CBF images in **q** were smoothed with a 3 × 3-pixel median filter. Time-series in **r** and **s** were plotted as the fraction (‘f’) with regard to *t* = 1 h. Scale bars, 500 μm (**a**,**b**) and 100 μm (**c**–**e**,**l**). H, high; L, low.

**Extended Data Fig. 10:**
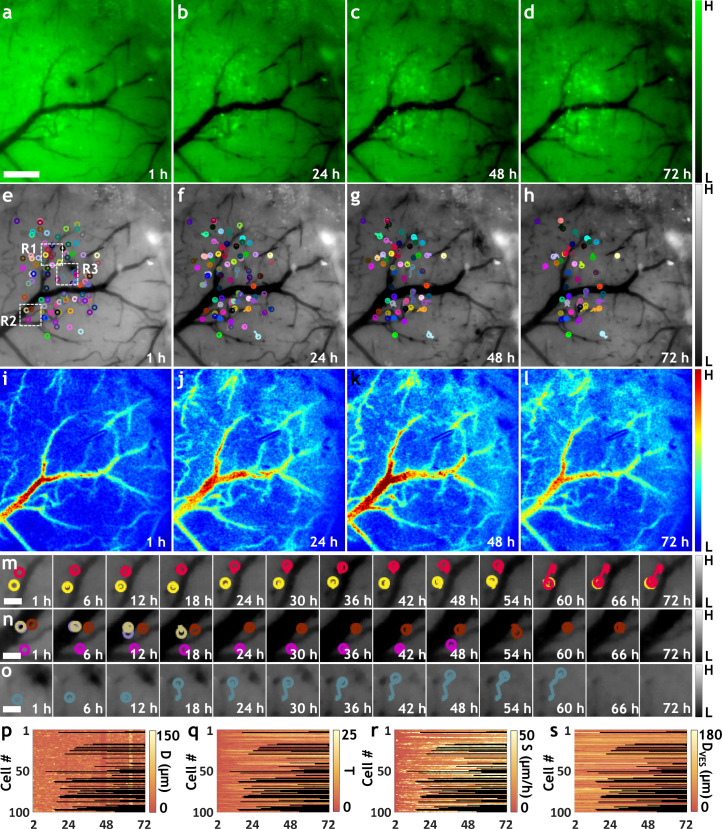
Neurosurveillance of brain tumor evolution in a second animal. (**a**-**d**) Wide-area maps of brain tumor progression over a 72-hour period in another brain tumor bearing mouse. (**e**-**h**) Migration trajectories of 100 fluorescent GL261-GFP murine glioma cells illustrated by pseudocolored traces overlaid on the corresponding IOS images showing the underlying microvasculature within the brain tumor microenvironment (BTME). Three regions of interest (ROIs, R1-R3) were identified for subsequent analyses. (**i**-**l**) Corresponding maps of cerebral blood flow (CBF). Magnified views of R1-R3, showing examples of glioma cells merging (**m**, R1); or intravasating into a pre-existing blood vessel (**n**, R2); or migrating to distant brain regions (for example > 100 μm) without interacting with other brain tumor cells or blood vessels, respectively. In vivo ‘functional assays’ of glioma cell migration during the TI phase, showing profiles of: (**p**) glioma cell migration (that is displacement from its location at start of imaging, ‘D’), (**q**) tortuosity of each migration trajectory (‘T’), (**r**) the speed (‘S’), and (**s**) the proximity to blood vessels (‘D_VES_’) for the ensemble of 100 murine glioma cells. Scale bar=500 μm, in (**e**) and scale bar=100 μm, in (**m-o**). H=high, L=low. Note: No images were acquired during post-inoculation hours 22, 44, and 45. Values for D, T, S and D_VES_ in (**p**), (**q**), (**r**) and (**s**) corresponding to these time points were linearly extrapolated from their nearest neighbors.

We harnessed neurosurveillance to characterize the cellular and vascular alterations that underlie brain tumor evolution (gliomagenesis) in *n* = 2 animals (M6 and M7). In contrast to in vitro cell migration assays that do not recapitulate the complexity of the in vivo brain tumor microenvironment (BTME), wide FoV (∼3 × 3 mm^2^) and high spatial resolution (~7 µm) neurosurveillance of brain tumor-bearing mice over 72 h enabled in vivo tracking of murine glioma cells within the heterogeneous BTME during the tumor initiation (TI) phase (Fig. 5a,b and Supplementary Video 12). For example, when we tracked *n* = 100 GL261 glioma cells tagged with green fluorescence protein (GFP) in vivo (Fig. 5b), we observed dynamic events (Supplementary Video 12) such as the merging of glioma cells (for example cells C1 and C2 in region R1; Fig. 5c); glioma cell intravasation into pre-existing blood vessels (for example cells C3 and C4 in region R2; Fig. 5d); and glioma cell motility (for example cell C5 in region R3; Fig. 5e). We were able to quantify the in vivo migratory dynamics of glioma cells in terms of their path tortuosity and speed (Fig. 5f–k). We observed that cells that interacted (for example cells C1/C2; Fig. 5f,i) or those that had similar fates (for example cells C3/C4; Fig. 5g,j) could exhibit substantially different migratory dynamics. Some glioma cells were also seen to migrate large distances (>100 μm) during the TI phase without merging with other glioma cells or intravasating into a pre-existing blood vessel (Fig. 5h,k). Characterizing the dynamics of the entire ensemble of glioma cells in terms of their displacement, migration tortuosity, speed and proximity to microvessels permitted us to phenotype these cells in vivo (Fig. 5l–o) analogous to measurements made with two-photon imaging^30^, but over an FoV that was >30× larger (for example 3 × 3 mm^2^ versus 0.5 × 0.5 mm^2^). We also phenotyped glioma cells in vivo in another animal, M7 (Extended Data Fig. 10). In addition, neurosurveillance permitted quantifying the attenuation of blood flow in pre-existing blood vessels (for example in region R4 in Fig. 5a, shown over a representative 24-h period in Fig. 5q,s) as glioma cells ‘co-opted’ and proliferated (Fig. 5p,r) within that perivascular niche (Supplementary Video 13).

**Fig. 6:**
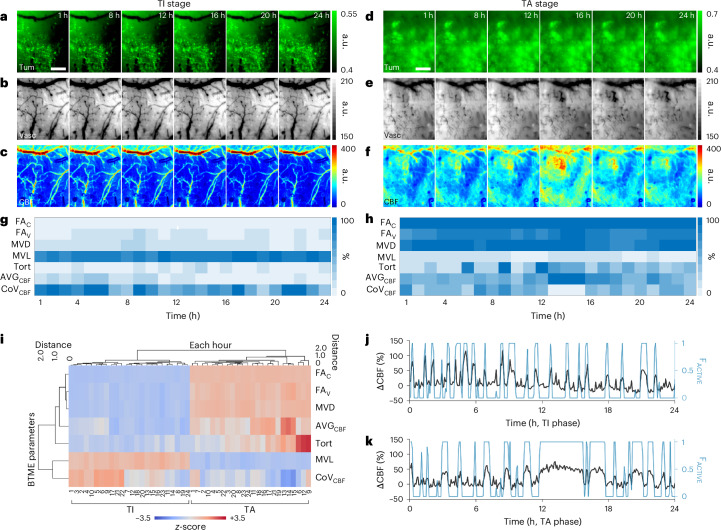
In vivo phenotyping of the brain tumor microenvironment during tumor initiation and tumor aggregation phases. **a**–**f**, Wide-area maps of tumor progression (**a**,**d**, ‘Tum’); microvasculature (**b**,**e**, ‘Vasc’); and CBF (**c**,**f**) during TI (**a**–**c**) and the subsequent TA (**d**–**f**) phases, respectively, over a representative 24-h period within a central 1.8 × 2.2-mm^2^ ROI (R5) from the FoV shown in Fig. 5a,b. All images are from mouse M6. FL images were divided by their IOS counterparts to minimize the impact of light absorption by CBV before their visualization in **a** and **d**. **g**,**h**, Panels summarizing microscopic (cellular: FA_C_), mesoscopic (vascular morphology: FA_V_, MVD, MVL and Tort) and macroscopic (functional: AVG_CBF_ and CoV_CBF_) characteristics of the BTME during TI (**g**) and TA (**h**) phases. FA_C_, fractional occupancy of the FoV by tumor cells; FA_V_, fractional occupancy of the FoV by blood vessels; MVD, mean vessel density; MVL, mean vessel length; Tort, vascular tortuosity; AVG_CBF_, average CBF; CoV_CBF_, temporal coefficient of variation of CBF. All parameters were computed on a ~100 × 100 μm grid (16 × 16-pixel grid) and the mean computed over the FoV at each time point. **i**, A double dendrogram illustrating our ability to discriminate the TI BTME from the TA BTME based on the micro-, meso- and macroscopic metrics measured in vivo during neurosurveillance. **j**,**k**, CBF (black) and behavioral activity (F_ACTIVE_, blue, computed every 5 min) time-series over a representative 24-h period for the TI (**j**) and TA (**k**) BTMEs. Mean CBF time-series were computed over the FoV (shown in **c**,**f**) over 5 min and normalized to the mean CBF during low activity (F_ACTIVE_ ≤ 0.5) epochs. The F_ACTIVE_ time-series is shown on the secondary *y* axis. Scale bar, 500 μm (**a**,**d**). a.u., arbitrary units. Panels **a**–**c**,**g**,**j** are from a representative 24-h period during D3 (during the TI phase). Panels **d**–**f**,**h**,**k** are from a representative 24-h period during D7 (during the TA phase).

Neurosurveillance of the same brain tumor-bearing mouse during the subsequent tumor aggregation (TA) phase enabled us to compare evolution of the BTME between TI and TA phases. The BTME during TI (shown over 24 h from post-inoculation days D2–D3 for region R5 marked in Fig. 5a,b and Supplementary Video 14), was characterized by glioma cell migration and co-option (Fig. 6a), stable microvessels (Fig. 6b) and regions of steady or attenuated CBF (Fig. 6c). In contrast, the BTME over the same brain region during the TA phase (shown over 24 h during D8–D9; Supplementary Video 15) was characterized by the aggregation of glioma cells (Fig. 6d) within a dense microvascular niche consisting of angiogenic vessels (Fig. 6e) and elevated CBF (Fig. 6f). The deterioration of the IOS channel’s signal-to-noise ratio (SNR) due to tumor-induced microvascular remodeling was consistent with previous reports^31^, although those studies did not contrast tumor growth phases as we did here. We then phenotyped these unique BTMEs based on microscopic (cellular), mesoscopic (vascular morphology) and macroscopic (functional) data continuously acquired in vivo with the CloudScope (Fig. 6g–h). The TI-phase BTME was characterized by minimal cell proliferation (low fractional area of glioma cells or FA_C_), minimal angiogenesis (low fractional area of vasculature or FA_V_), stable microvessel density (MVD), microvessel length (MVL), microvessel tortuosity (Tort) and mean CBF (AVG_CBF_) and temporally heterogeneous CBF (CoV_CBF_) (Fig. 6g). In contrast, the TA-phase BTME was characterized by glioma cell proliferation (elevated FA_C_) supported by an expanding vascular niche with robust angiogenesis (elevated FA_V_, MVD and Tort), elevated AVG_CBF_, and a low CoV_CBF_ (Fig. 6h). Finally, we characterized the dynamics of glioma evolution in vivo (Supplementary Video 16). Crucially, these multiscale in vivo neurosurveillance data enabled differentiation of the TI-phase BTME from the TA-phase BTME (Fig. 6i). Moreover, despite the disruption of the neurovascular unit by glioma cells in the TA-phase BTME (Supplementary Fig. 10), CBF changes during this phase remained correlated with animal activity analogous to that during the TI phase (Fig. 6j,k; R = 0.80 and 0.90 between CBF time-series and F_ACTIVE_ for TI versus TA, respectively). Collectively, the ability of neurosurveillance to interrogate critical aspects of the brain tumor lifecycle makes the CloudScope a powerful tool for understanding the early steps in gliomagenesis, testing therapies and developing clinical biomarkers predicated on in vivo glioma cell-BTME interactions.

## Discussion

The characterization of neurovascular changes that accompany the initiation, progression and culmination (that is, lifecycle) of preclinical CNS disease models necessitates a multicontrast imaging system that can be continuously operated over multiple days. CloudScope enabled autonomous in vivo neuroimaging for 24 h or longer, in freely behaving animals. We successfully conducted neurosurveillance of critical in vivo neurophysiological variables such as Neu_ACT_, CBF, CBV, Hb_SAT_ and cell tracking. This multicontrast capability made image-based phenotyping of the CNS disease microenvironment possible across cellular/neuronal (microscopic), vascular/hemodynamic (mesoscopic) and tissue (macroscopic) spatial scales. Additionally, the operation of multiple CloudScopes in parallel is ideal for high-throughput applications such as drug screening by pharmaceutical companies or contract research organizations (CROs). The use of hobbyist electronics, open-source software and 3D-printed materials ensured that the CloudScope was affordable for users and accessible to under-resourced research and educational environments.

The acquisition of 24-h neurosurveillance data enabled us to test the feasibility of predicting an animal’s behavior from its Neu_ACT_ recordings using a DL framework. We successfully predicted the behavioral state of the animal using a combination of conventional ResNet50 and BiLSTM modules, the performance of which was visualized via Grad-CAM. One can envision expanding this neurosurveillance-informed DL framework to include a more powerful ‘transformer’-based architecture^32^ for behavioral classification. Our ability to achieve high average per-class prediction accuracies (>75%) for each animal by using only that animal’s data (without pooling with data from other animals) to train and predict its behavior, establishes the basis for developing a new generation of DL pipelines to tease out individual-specific traits; however, it should be noted that prediction accuracies depend on the amount of the training data available for each Behavior_SYL_. For example, the lower accuracy (64 ± 15%) for animal M5’s R syllable compared to its MM syllable (94 ± 8%) was likely a result of the relatively low number of R syllables observed for M5 during the 24-h imaging period. One could circumvent this by imaging for longer or merging infrequent Behavior_SYL_ before employing them in the DL pipeline. Neurosurveillance-informed DL models could also be used to elucidate learning mechanisms under healthy or disease conditions such as during rehabilitation following stroke or in individuals with Parkinson’s disease. Cross-modality use of neurosurveillance-informed DL, for example when predicting patterns of CBF changes from Neu_ACT_, could help us better understand neurovascular dynamics and its disruption in different CNS neuropathologies. It may also be prudent to explore DL-interpretability techniques such as Grad-CAM^18^ or LIME^33^ to better relate DL-based features to their neurobiological underpinnings. We believe that 24-h multicontrast neurosurveillance and behavioral data in conjunction with other well-established DL pipelines, such as the CEBRA framework^34^, could expand the CloudScope’s impact and utility for CNS disease and neuroscience applications.

Next, we applied neurosurveillance to explore the complex interplay between Neu_ACT_ and cerebral hemodynamics in freely behaving animals under healthy and disease conditions, including seizures and brain tumors. Our ability to visualize and characterize neurovascular dynamics for more than 24 h could prove invaluable for understanding the causes, consequences, and recurrence of seizures. Neurosurveillance could also be used for screening anti-seizure drugs, as well as titrating their dose and timing for optimal efficacy. As neurosurveillance enabled multiscale phenotyping of the dynamic BTME, one can envision using it in combination with radiation therapy or chemotherapeutic agents such as berberine that are known to arrest tumor cell migration^35^, to customize treatment and optimize efficacy (that is, precision medicine approaches). We also used neurosurveillance to identify perivascular niches, a key harbor of brain tumor stem-like cells, by identifying microvessel territories co-opted by glioma cells. Imaging these niches during treatment with antiangiogenic or anti-co-optive drugs could help elucidate the mechanisms underlying the development of resistance to such therapies^36^. Collectively, these data illustrate the power of neurosurveillance for a range of CNS pathologies, and the potential of CloudScope to transform our understanding of the disease microenvironment and catalyze the development of more efficacious therapeutics and in vivo biomarkers.

We envision several exciting developments to further enhance CloudScope’s utility. For instance, modifying its design to acquire images over a wider FoV (for example 8 × 10 mm^2^ (ref. ^37^)) would enable neurosurveillance of the entire murine cortex. DL methods (for example DeepWonder^38^) could be applied to resolve the Neu_ACT_ time-series of individual neuronal soma from CloudScope images. Employing an ultra-low-power image sensor that supports high-speed image acquisition would permit neurosurveillance of faster neurovascular events. Including an additional IOS channel at ~590 nm could help improve the signal-to-noise ratio (SNR) of measurements of intravascular oxygen saturation. Implementing an additional blue, red or near-infrared FL channel would enable a host of new neurosurveillance applications such as imaging neuron-astrocyte crosstalk, identifying neuron–glioma interactions during brain tumor evolution and testing the efficacy of new tissue-engineered constructs to promote healing of cranial bone defects. CloudScope could also be paired with an electrowetting lens^2^, allowing convenient focusing during long-term experiments. Incorporating miniaturized EEG or neurochemical sensors would further expand CloudScope’s neurosurveillance capabilities. Neurosurveillance could also be combined with recordings from multiple behavioral cameras and advanced behavioral analysis tools such as DeepLabCut^39^, to gain an in depth understanding of the Neu_ACT_–behavior nexus. When combined with interventions such as implantable drug infusion pumps or optogenetic stimulation, the power of neurosurveillance could be harnessed to screen potential drug candidates and fine tune their efficacy. Designing a hermetic seal or sterilization protocol for CloudScopes would enable the neurosurveillance of infectious brain disease models such as *Mycobacterium* *tuberculosis*^40^.

One could enhance the CloudScope’s Wi-Fi bandwidth by using either the Raspberry Pi’s Ethernet port or a personal Wi-Fi hotspot. High-bandwidth internet accessibility together with cloud-based storage could ‘democratize’ the study of CNS disease and neuroscience by providing researchers around the world unrestricted access to in vivo imaging experiments and data. Embedded real-time processing by exploiting the Raspberry Pi’s CPU cores could enable the CloudScope to automatically recognize when preset criteria such as Neu_ACT_ thresholds or behavioral stimuli are met, and switch between ‘sequential’ and ‘livestream’ modes, enabling a ‘smart’ neuroimaging paradigm. Finally, our web-based architecture enables ‘time-shared’ multimodal imaging, which potentially reduces animal use and waste by repurposing the same animals for multiple in vivo imaging experiments. Given its extant capabilities and future enhancements, we believe that CloudScope-based neurosurveillance has the potential to foster global neuroscientific collaborations, reduce the entry barriers to research and enable new studies of the brain in health and disease.

## Methods

### System architecture and software platform

Figure 1a illustrates the overall cloud-based IoT architecture. Each CloudScope was paired with a behavioral camera. To create a bank of CloudScopes with behavioral cameras for neurosurveillance of multiple animals in parallel (Extended Data Fig. 1a–d), we implemented multiple cloud-based servers using a ‘LightSail’ instance of the AWS platform. Each LightSail instance controlled one CloudScope and behavioral camera. Custom software was written in the software language Python to create a GUI for controlling image acquisition (Extended Data Fig. 1e–g). The GUI’s main window (Extended Data Fig. 1e) was used to select an illumination source, set its brightness, specify image acquisition parameters such as camera exposure time and gain and acquire images in the ‘livestream’ mode. The user could open a secondary window to specify the parameters for initiating a ‘sequential stream’ mode (Extended Data Fig. 1f), wherein all illumination sources were repeatedly switched ON/OFF, and images acquired over multiple user-specified ‘cycles’. Images were formed via the passage of light through an aspheric lens (focal length = 4.6 mm, A390-A, Thorlabs) and a long-pass filter (cutoff wavelength = 510 nm, Omega Optical) and recorded on a CMOS image sensor (OV5647, OmniVision Technologies).

### Control module

The control module was located above the cage and connected to the CloudScope via a ~1-m FFC and 32-gauge wires (Fig. 1b). It consists of a Raspberry Pi 4B processer Pi (Raspberry Pi), a Teensyduino 4.1 microcontroller (PJRC.com) and analog circuitry for powering the CloudScope’s illumination sources (Supplementary Fig. 3). A Python script running on the Raspberry Pi managed all communications with the cloud-based server by connecting to the internet via local Wi-Fi. A USB drive attached to one of the Raspberry Pi’s USB 2.0 ports was used to store all acquired images locally. In parallel, the Raspberry Pi communicated via the FFC cable connected to its camera serial interface, with an off-the-shelf flexible PCB (B0066-02 NoIR, ArduCam) that controlled the CMOS image sensor. All commands for setting image sensor-related parameters (for example exposure time, analog and digital gain) and image data were transferred via this interface. Control of the illumination parameters was delegated to the Teensyduino 4.1. To achieve this, the Raspberry Pi and the Teensyduino were used in an ‘initiator–responder’ configuration wherein the Teensyduino listened for commands from the Raspberry Pi via its UART port. Once information specifying an illumination source and its brightness was received from the Raspberry Pi, the Teensyduino used its pulse width modulation (PWM) modules (PMW1 and PMW2 modules denoted in Supplementary Fig. 3 for FL and GR illumination) or its I^2^C module (for LS illumination) to switch ON/OFF the appropriate illumination source. The PWM modules used a standard load bearing circuit with an NPN transistor and a current limiting resistor (R_LIM_ in Supplementary Fig. 3) to drive the FL and GR LEDs. In contrast, the I^2^C module communicated with a 12-bit digital-to-analog converter (MCP4725, Texas Instruments) that created an analog voltage (V_LS_ in Supplementary Fig. 3). V_LS_ was converted to the appropriate current amplitude for driving the laser diode (a vertical cavity surface emitting laser, 680S or V00013, Vixar or LV670, Thorlabs) via an operational amplifier (OPA, TLV2462, Texas Instruments) based constant-current circuit. The resistor R_s_ was used to set the proportionality constant for voltage to current conversion.

### Cranial window preparation

All animal procedures were approved by the Johns Hopkins University Animal Care and Use Committee. For healthy and seizure imaging (animals M1–M5), craniotomy-based cranial windows (centered at AP = −2 mm; ML = 2 mm in relation to the bregma, diameter = 3 mm) were prepared on 2–3-month-old female (*n* = 3) or male (*n* = 2) C57BL/6J mice (Jackson Laboratories). Mice were anesthetized with 1–2% isoflurane and placed in a stereotaxic frame (Benchmark, Myneurolab.com). A subcutaneous injection of dexamethasone (0.1 mg in 0.05 ml solution) was administered before surgery to prevent brain swelling and lidocaine (0.5 ml, 2% w/v) injected under the scalp to alleviate pain. The skull was exposed with a midline scalp incision and a circular piece of bone over the right hemisphere (3-mm diameter with its center at the stereotaxic coordinates AP 2 mm; ML 2 mm, approximately over the murine sensorimotor cortices) was removed using a hand-held dental drill (Foredom) with a 0.7-mm drill bit (Finescience 19007-07). AAV9.CAG.GCaMP6s.WPRE.SV40 virus (Addgene) was injected at six evenly spaced sites within the craniotomy at a depth of 0.5 mm using a thin glass pipette and a Nanoject II (Drummond Scientific) injector. Each injection contained 40 nl of virus at a titer of 5 × 10^12^. Next, as shown by the schematic in Supplementary Fig. 4, a circular, 3-mm diameter cover slip was placed over the craniotomy and glued in place using Vetbond tissue adhesive. Next, a 3D-printed head-mount for attaching the CloudScope was affixed to the skull using cyanoacrylate glue and dental cement. Following surgery, mice received subcutaneous injections of Baytril (2.5 mg kg^−1^) to prevent infections and buprenorphine (0.3 mg ml^−1^) to alleviate pain. A cranial window was made on a sixth animal that did not survive and was excluded from subsequent analyses. For brain tumor imaging (in mice M6–M7), via a similar procedure, a 4-mm diameter cranial window was made centered at AP = 3 mm; ML = 3 mm in relation to the intersection of the lambdoid and the sagittal sutures in *n* = 2 adult >2-month-old female C57BL/6J mice (Jackson Laboratories) and fluorescent GL261–GFP murine glioma cells were injected into the brain parenchyma (50,000 cells inoculated at 0.2 mm and 0.5 mm depth in M6, or 100,000 cells inoculated at 0.5 mm and 1.0 mm depth in M7. Animals were killed well before any appreciable tumor burden was established according to the protocol.

### Seizure induction and scoring seizure severity

Seizures were induced in via an i.p. injection of PTZ (40 mg kg^−1^ body weight), a GABA_A_ antagonist^41^ in unanesthetized CloudScope-bearing mice (duration of injection = ~1 min). The severity of the seizures was characterized by a ‘seizure score’ based on a modified Racine scale^28^.

### In vivo imaging experiments

Neurosurveillance of healthy and seizure-stricken brain activity was performed in *n* = 5 animals (M1–M5). Each animal was habituated and subsequently imaged for two 24-h periods: one for imaging healthy brain function, and another for imaging seizure-induced neurovascular changes. A seizure was induced ~2–3 h after the start of image acquisition on the second day via i.p. injection of PTZ. Mouse M5 did not survive the seizure. Neurosurveillance was performed on *n* = 2 animals (M6 and M7) to characterize changes in a BTME. Both animals were allowed to recover for > 1 h after cranial window surgery and brain tumor inoculation (D0) and were habituated to the CloudScope <24 h prior to image acquisition. In mouse M6, neurosurveillance was performed for ~5 days (D1–D5, with intermittent breaks), the mouse rested for ~2 days (D6–D7), and neurosurveillance conducted for another ~1 day (D8–D9) before killing. The in vivo behavior of fluorescent GL261–GFP glioma cells was observed via the CloudScope’s FL channel to identify TI (D1–D4), and TA phases (D8–D9). For animal M7, neurosurveillance was conducted for ~4 days (D1–D5, with intermittent breaks) before killing. Imaging timelines are summarized in Supplementary Fig. 11. To enable imaging of the early phases of gliomagenesis, it was necessary to shorten the habituation period. In the future, one could circumvent this limitation by employing a ‘resealable’^42^ or ‘soft’/polydimethylsiloxane (PDMS)-based^43^ cranial window to enable inoculation of brain tumor cells after extensive habituation. Finally, high-speed (~19 fps) image acquisition was conducted for ~10 min for animal M4. All experiments were performed with the CloudScope.

### Acquisition of FITC–dextran tracer kinetics

FITC-Dextran (100 µl, 10 mg ml^−1^, 250 kDa, Sigma-Aldrich) was administered intravenously under isoflurane anesthesia via the animal’s tail vein^23^. FL images were acquired in the livestream mode (exposure = ~50 ms, 512 × 512 pixels), registered offline using ImageJ’s MultiStackReg and TurboReg plugins^44^ and resampled at 100 ms time steps using a customized MATLAB (MathWorks) script. Arteries and veins within the FoV were identified based on tracer arrival times (reaching 50% of maximum intensity) for each vessel segment.

### Continuous image acquisition for neurosurveillance

Images were acquired with the CloudScope every ~5 s for healthy and seizure experiments, or 30 s for BTME experiments under blue (FL, with ~453 ± 20 nm excitation), GR (~530 ± 20 nm) and LS (670 nm or 680 nm) illumination. See Supplementary Table 3 for details. Notably, delays (‘OFF’, shown in Supplementary Fig. 5) were inserted to prevent the sensor from overheating during acquisitions that were ≥24 h. While the FL- and GR-channel images were acquired as 512 × 512-pixel images, the LS images were acquired as 1,536 × 1,536-pixel images to preserve the spatial pattern of laser speckles. Imaging parameters were set at the beginning of an experiment and kept constant during the ensuing 24 h. In contrast, the animal’s behavior was recorded via the behavioral camera (B0066-02 NoIR, ArduCaM) controlled by a second Raspberry Pi at ~4 fps as either 512 × 512 or 256 × 256-pixel images. A heat sink attached to the behavioral camera prevented overheating of the image sensor. Time stamps with millisecond temporal resolution accessible via the universal coordinated time (UTC) clock settings maintained by the Raspberry Pi were included as part of the image filename to identify the acquisition time.

### Circumventing wireless network instabilities

Although the CloudScope stored all the acquired data on a local USB drive, it relied on Wi-Fi for communications with the remote server. Therefore, CloudScope operation was susceptible to fluctuations in Wi-Fi traffic. To address this, we incorporated an ‘emergency sequence’ into CloudScope’s software so that in the event of a transient loss of internet connectivity, it continued to save data on the local USB drive. We also encountered instances during which initiation of the emergency sequence failed, which resulted in ~45 min of lost data during one of the 24-h experiments. We resolved this by using a personal Wi-Fi hotspot instead of the institutional Wi-Fi network to control/stream CloudScope data.

### Immunohistochemistry

The BTME vasculature was labeled with TRITC-dextran-gelatin (155 kDa, Sigma-Aldrich) injected via transcardiac perfusion. Additionally, either astrocytes (GFAP) or vascular endothelia (laminin) or blood–brain barrier (BBB) (GLUT-1) were labeled ex vivo in the near-infrared (NIR) channel, using polyclonal rabbit anti-GFAP mouse antibody (Agilent Dako), rabbit α-laminin antibody (Sigma-Aldrich), and a monoclonal rabbit GLUT-1 antibody (Cell Signaling Technology), respectively. Goat anti-rabbit IgG Alexa Fluor 647 (Thermo Fisher Scientific) was used as a secondary antibody. Sections were imaged at ×20 on a multichannel Zeiss Axio Imager M2 fluorescence microscope.

### Computation of CBF maps

Maps of CBF were computed in a.u. using a 11 × 11-pixel neighborhood (N) as described elsewhere^5^. Of note, CBF maps can be created by computing speckle contrast using different pixel neighborhoods in either the spatial, temporal or spatiotemporal domains^5^. Choosing a spatial neighborhood maximizes temporal resolution at the expense of spatial resolution. Conversely, choosing a temporal neighborhood maximizes spatial resolution at the expense of temporal resolution, with the neighborhood size impacting overall image quality (Supplementary Fig. 12 and Supplementary Video 17). Neighborhoods for computing speckle contrast were applied with a sliding step size of 1 pixel, which resulted in adjacent pixels having dependent CBF values. While a step size similar to the size of the speckle contrasting neighborhood could circumvent this issue, it would result in pixelated-looking CBF maps. Additionally, the CBF image stack was normalized to the average CBF computed for a reference 30-min ‘baseline’ (‘BL’) period to yield an image stack of fractional CBF value (CBF_frac_).

### Resampling in the time domain

Image stacks for each channel were linearly resampled to a temporal resolution of 5 or 30 s via a MATLAB script. In addition, LS and CBF images were resampled using a 3 × 3-pixel kernel so that their spatial resolution matched the 512 × 512-pixel resolution of the images acquired from the FL and IOS channels and used for all subsequent analyses.

### Image co-registration

Image stacks were manually inspected for motion (Supplementary Fig. 13 shows a quantification) and images were co-registered via ImageJ’s MultiStackReg/TurboReg^44^ plugins.

### Spatial resampling to minimize residual misalignment

All image stacks (FL, GR, LS and CBF) were spatially resampled with a nonoverlapping 8 × 8-pixel spatial kernel, resulting in 64 × 64-pixel images, which reduced the effect of residual misalignments (~<8 × 8 pixels or ~50 × 50 μm) that were not corrected during the image co-registration step. Speckle patterns were not visible at this scale in the LS images. These 64 × 64-pixel image stacks were used for all analyses in healthy and seizure experiments unless otherwise noted.

### Computing Neu_ACT_

First, we used the modified Beer–Lambert law^45^, and modeled the light intensity recorded at each pixel of the FL image as follows:

1 $${\mathrm{FL}}=\left\{{{{\alpha }} \mathrm{Neu}}_{\mathrm{ACT}}+\mathrm{BG}\right\}{{\rm{I}}}_{\mathrm{ILLU}} \exp \left({-A}\right)$$

Here, *α* is a scalar representing the amount of neuropil/neuronal GCaMP6s expression in each pixel. The GCaMP6s expression is expected to be nonuniform over the 3 × 3-mm^2^ FoV because it arises from the local delivery of a viral vector. Neu_ACT_ represents the fluorescence from Ca^2+^ bound GCaMP6s in neuropils or neuronal somata. BG: background fluorescence in that pixel from sources unrelated to Neu_ACT_. For simplicity, we assume this to be a constant across time. I_ILLU_: the corresponding excitation light intensity. A: light absorption by hemoglobin in that pixel. In addition, the absorption of excitation and emission light by oxy- and deoxy-hemoglobin were represented as one term.

Therefore, to extract Neu_ACT_ from FL intensity in any pixel, we divided its FL time-series by its mean intensity during a reference period and computed a fractional FL intensity (FL_frac_)^4^:

2 $${\mathrm{FL}}_{\mathrm{frac}}=\frac{\mathrm{FL}}{{\mathrm{FL}}_{{\rm{0}}}}=\frac{\left\{{{{\alpha }} \mathrm{Neu}}_{\mathrm{ACT}}+\mathrm{BG}\right\} {{\rm{I}}}_{\mathrm{ILLU}} \exp \left({-A}\right)}{\left\{{{{\alpha }} \mathrm{Neu}}_{\mathrm{ACT},\,0}+\mathrm{BG}\right\} {{\rm{I}}}_{\mathrm{ILLU},\,0} \exp \left({-A_{0}}\right)}$$

Here, the subscript ‘0’ denotes the mean during the reference period. Next, by assuming a constant input illumination, we can reduce equation (2) to:

3 $${\mathrm{FL}}_{\mathrm{frac}}=\frac{\left\{{{{\alpha }} \mathrm{Neu}}_{\mathrm{ACT}}+\mathrm{BG}\right\}}{\left\{{{{\alpha }} \mathrm{Neu}}_{\mathrm{ACT},{\rm{0}}}+\mathrm{BG}\right\}} \exp \left[-\left({A-{A}_{0}}\right)\right]$$

Here, the exponential term refers to the incremental light absorption by hemoglobin, for which we used the GR time-series as a surrogate. To remove the effects of non-uniform illumination, the GR time-series was also normalized (GR_frac_) by dividing it by its mean intensity (GR_0_) during the reference period:

4 $${\rm{GR}}_{\rm{frac}}=\frac{\rm{GR}}{{\rm{GR}}_{0}} \sim \exp \left[-\left({A-{A}_{0}}\right)\right]$$

Next, the hemodynamic correction was performed as follows^4^:

5 $${{\mathrm{FL}}_{\mathrm{frac}}}^{* }=\frac{{\mathrm{FL}}_{\mathrm{frac}}}{{\mathrm{GR}}_{\mathrm{frac}}}=\frac{\left\{{{\rm{\alpha }} \mathrm{Neu}}_{\mathrm{ACT}}+\mathrm{BG}\right\}}{\left\{{{\rm{\alpha }} \mathrm{Neu}}_{\mathrm{ACT},{\rm{0}}}+\rm{BG}\right\}}$$

As the term ‘α∙Neu_ACT,0_ + BG’ only changes in the spatial domain, we defined a new constant β:

6 $$\beta ={{\rm{\alpha }} \mathrm{Neu}}_{\mathrm{ACT},{\rm{0}}}+\rm{BG}$$

Now, equation (6) can be rewritten as:

7 $${{\mathrm{FL}}_{\mathrm{frac}}}^{* }=\frac{{\rm{\alpha }}}{\beta } {\mathrm{Neu}}_{\mathrm{ACT}}+\frac{\mathrm{BG}}{\beta }$$

And simplified to:

8 $${{{\rm{FL}}}_{{\rm{frac}}}}^{* }=\gamma {{\rm{Neu}}}_{{\rm{ACT}}}+{\rm{BG}}^{\prime}$$

Here, $${\rm{\gamma }}=\,{\rm{\alpha }}/{\rm{\beta }}$$ and $${\rm{B}}{{\rm{G}}}^{{\prime} }={\rm{BG}}/{\rm{\beta }}$$.

Note: both *γ* and BG′ are spatial variables and represent the nonuniform expression of neuropil/neuronal GCaMP and the background signal, respectively. Therefore, to correct the effects of γ and BG′, we transformed FL_frac_* into a *z*-score. To preserve the magnitude of the activity related Neu_ACT_ changes, the *z*-score transformation employed the mean and s.d. of the FL_frac_* time-series during MM periods. To do so, we first defined the following:

9 $${{{\mu }}}_{\mathrm{MM}}=\frac{1}{{{{N}}}_{\mathrm{MM}}} \mathop{\sum }\limits_{\rm{MM}}{{\mathrm{FL}}_{\mathrm{frac}}}^{* }={{\gamma }} {\mu }_{\mathrm{MM},\,{\mathrm{Neu}}_{\mathrm{ACT}}}+{\mathrm{BG}}^{{\prime} }$$

10 $${\sigma}_{\mathrm{MM}}=\frac{1}{{N}_{\mathrm{MM}}} \sqrt{\mathop{\sum }\limits_{\mathrm{MM}}{\left({{\mathrm{FL}}_{\mathrm{frac}}}^{* }-{{{\mu }}}_{\mathrm{MM}}\right)}^{2}}={\gamma}{\sigma}_{{\mathrm{MM}},\,{\mathrm{Neu}}_{\mathrm{ACT}}}$$

Here, *N*_MM_ and ${\sum }_{MM}()$ denote the number of time points the animal spent in the MM state and the summation of FL_frac_* during 24 h of imaging.

Then, we used μ_MM_ and σ_MM_ to transform FL_frac_* into a *z*-score that indicated GCaMP-bound neuropil/neuronal Ca^2+^ activity that was unbiased by background signals and nonuniform GCaMP expression:

11 $${{\rm{Neu}}_{\rm{ACT}}}\left(Z\right)=\frac{{{\rm{FL}}_{\rm{frac}}}^{* }-{\mu }_{\rm{MM}}}{{\sigma }_{\rm{MM}}}$$

Here,

12 $${{\mathrm{Neu}}}_{\mathrm{ACT}}\left({{Z}}\right)=\frac{{\rm{\gamma }} {\mathrm{Neu}}_{\mathrm{ACT}}+{\mathrm{BG}}^{{\prime} }-{\rm{\gamma }} {{\rm{\mu }}}_{\mathrm{MM},\,{\mathrm{Neu}}_{\mathrm{ACT}}}-{\mathrm{BG}}^{{\prime} }}{{\rm{\gamma }}\, {{\rm{\sigma }}}_{\mathrm{MM},\,{\mathrm{Neu}}_{\mathrm{ACT}}}}$$

Thus,

13 $${{{\rm{Neu}}}}_{{\rm{ACT}}}\left({{Z}}\right)=\frac{{{\rm{Neu}}}_{{\rm{ACT}}}-{{\rm{\mu }}}_{{\rm{MM}},\,{{\rm{Neu}}}_{{\rm{ACT}}}}}{{{\rm{\sigma }}}_{{\rm{MM}},\,{{\rm{Neu}}}_{{\rm{ACT}}}}}$$

### Computing hemodynamic parameters

Following^4^, GR and LS image stacks were used to compute changes in oxy- ($\Delta$HbO), deoxy (ΔHb) and total (ΔHbT) hemoglobin content. Intravascular oxygen saturation (Hb_SAT_) was estimated using these values and assuming baseline HbT and Hb_SAT_ of 100 μM and 60%^46^, respectively.

### Generation of global time-series

Global time-series for each neurophysiological variable were generated by computing its spatial average over the brain parenchyma at each time point.

### Extraction of vasomotor dynamics from IOS images

Using the modified Beer–Lambert law^45^, one can write:

14 $${\mathrm{ln}}\,{\rm{GR}}={\mathrm{ln}}\,{{\rm{I}}}_{\rm{ILLU}}-\left[{\varepsilon }_{\mathrm{Hb}} {{{C}}}_{\mathrm{Hb}}\left({{x}},{{y}}\right)+{\varepsilon }_{\mathrm{HbO}} {{{C}}}_{\mathrm{HbO}}\left({{x}},{{y}}\right)\right] \mathrm{PL}-{{G}}$$

Here, GR and I_ILLU_ are intensities of the measured and illumination light in a given pixel, respectively, during IOS imaging under GR illumination. *C*_Hb_ and *C*_HbO_ are concentrations of deoxy- (Hb) and oxy-hemoglobin (HbO) at that location, and *ε*_Hb_ and *ε*_HbO_ their mean light extinction coefficients in the given wavelength range (~530 nm). G is a constant. As, ~530 nm is an isosbestic wavelength for light absorption by oxy- and deoxy-hemoglobin, we can simplify equation (14) to:

15 $${\mathrm{ln}}\,{\rm{GR}}={\mathrm{ln}}\,{{\rm{I}}}_{\rm{ILLU}}-{\varepsilon }_{\mathrm{HbT}} {{{C}}}_{\mathrm{HbT}} \mathrm{PL}-{{G}}$$

Here, *ε*_HbT_ and *C*_HbT_ denote the extinction coefficient and concentration for total hemoglobin content (HbT). Next, as shown in Supplementary Fig. 9, one could apply equation (15) for a background region (‘BG’) in the vicinity of a given vessel segment as:

16 $${\mathrm{ln}}\,{\rm{GR}_{{BG}}}={\mathrm{ln}}\,{{\rm{I}}}_{\rm ILLU,\mathrm{BG}}\,-{\varepsilon }_{\mathrm{HbT}} {{{C}}}_{\mathrm{HbT},\mathrm{BG}}{\rm{PL}}_{\rm{BG}}-{{G}}$$

Similarly, one could assume that the minimum intensity (GR_MIN_) occured where light traverses a vessel (‘Ves’), and apply equation (15) as:

17 $${\mathrm{ln}}\,{{\rm{GR}}}_{\mathrm{Min},\mathrm{Ves}}=\mathrm{ln}\,{{\rm{I}}}_{\rm{ILLU},\mathrm{Ves}}-{\varepsilon }_{\mathrm{HbT}} {{{C}}}_{\mathrm{HbT},\mathrm{Ves}}{\mathrm{PL}}_{\mathrm{Ves}}-{{G}}$$

I_ILLU_ levels over the vessel and background regions can be assumed to be approximately equal if the background was chosen near the vessel, that is, I_ILLU,Ves_ = I_ILLU,BG_. Moreover, as described previously^47^ one could relate the optical path lengths to the vessel diameter as:

18 $${{\rm{PL}}}_{{\rm{Ves}}}={{\rm{D}}}_{{\rm{Ves}}}+{{\rm{PL}}}_{{\rm{BG}}}$$

Here, D_Ves_ denotes the vessel diameter. By combining equations (14)–(18), one can write:

19 $${\mathrm{ln}}\frac{{{\rm{GR}}}_{\mathrm{BG}}}{{{\rm{GR}}}_{\mathrm{MIN},\mathrm{Ves}}}={\varepsilon }_{\mathrm{HbT}} {{{C}}}_{\mathrm{HbT},\mathrm{Ves}} {{D}_{{Ves}}}$$

Treating ‘*ε*_HbT,Ves_*C*_HbT_’ as a constant for each vessel segment by assuming a stable hematocrit, the vessel diameter at any given time point ‘*t*’ can be expressed as a fraction of the mean vessel diameter during a reference period at the beginning of image acquisition.

### Additional processing for visualization purposes

Hb_SAT_ images in Extended Data Figs. 7 and 8, Supplementary Fig. 14b and Supplementary Videos 7 and 9–11, are shown following the application of a nonlocal means filter using a ~300 × 300 μm (51 × 51-pixel) neighborhood. Before its application the nonlocal means filter was also validated on a separate dataset for its capacity to preserve temporal changes in Hb_SAT_ data (Supplementary Fig. 14).

CBF maps shown in Extended Data Figs. 4b, 7a,d and 8a–c and Supplementary Videos 7 and 9–11 are smoothed using the formula:

20 $${\mathrm{CBF}}_{\mathrm{DENOISED}}=\frac{{\left\langle {\mathrm{CBF}}_{{\rm{O}}}\right\rangle }_{N\times N}}{{\left\langle {\mathrm{CBF}}_{\mathrm{REF}}\right\rangle }_{N\times N}}\bullet {\mathrm{CBF}}_{\mathrm{REF}}$$

Here, CBF_O_ and CBF_DENOISED_ are the original and denoised CBF images. CBF_REF_ is the mean CBF map computed over a 1-min pre-seizure period (for the seizure experiments) and a 30-min baseline period (for the 24-h nonseizure experiments). <>_*N* × *N*_ denotes the spatial average computed over an *N* × *N*-pixel neighborhood (*N* = 17 pixels or ~100 μm).

### Annotating and quantifying animal behavior

Videos of animal behavior were analyzed offline and each animal’s behavior manually identified as belonging to one of three ‘syllables’^8^: MM (the animal was at rest), R and INT (instances when the animal was active but not running).

### Deep learning-based behavioral prediction

Our DL pipeline (Extended Data Fig. 5a) consisted of four phases: (1) data preparation; (2) the DL model; (3) model training and performance evaluation; and (4) model interpretation.

#### Data preparation

For each mouse, we extracted Neu_ACT_ images over 1-min periods (Neu_ACT_ ‘blocks’) during which the animal’s Behavior_SYL_ (MM, INT or R) remained unchanged. To circumvent model bias, we chose a random subset of Neu_ACT_ blocks corresponding to each of the two dominant Behavior_SYL_ (MM and INT) that were comparable in their occurrence to the R syllable (Supplementary Table 4). Next, we set aside 10% of this dataset as ‘test’ data and split the remainder into ‘training’ and ‘validation’ datasets via a ‘stratified shuffle split’ approach that permitted maintaining the original class distributions in the split data (Supplementary Table 5). Subsequently, training and validation datasets were fed into the DL pipeline over a maximum of 50× iterations as described elsewhere^48^.

#### The DL model

The DL model consisted of a ResNet50 (pretrained on ImageNet data^49^) and a BiLSTM architecture. The final FC layer of the ResNet50 was removed and its last convolutional layer utilized as a set of feature maps (FMs, *n* = 2,048 maps, with 7 × 7 pixels in each map), which was vectorized and input into the BiLSTM module to capture the temporal evolution of features across constituent images of each Neu_ACT_ block^48^. Next, we added a single FC layer at the end of the BiLSTM output. The FC layer used the BiLSTM outputs to produce three ‘class scores’ per Neu_ACT_ block, with each score describing the likelihood of a given Behavior_SYL_ (MM, INT or R) being associated with it. The syllable with the highest class score was chosen as the predicted Behavior_SYL_ via a SoftMax function.

#### Model training and performance evaluation

Images within each Neu_ACT_ block were resized (to 224 × 224-pixel images for compatibility with the pretrained ResNet50) and all weights in the DL model trained end-to-end for a maximum of 50× iterations (batch size = 32× Neu_ACT_ blocks) via an Adam optimizer that implemented a cross-entropy loss function^50^. Fivefold validation was conducted, wherein we independently trained and tested the DL model using five random repetitions of the data preparation step. Supplementary Table 5 provides a summary of the hyper-parameters.

#### Model interpretation

As described previously^18^, we used Grad-CAM (Extended Data Fig. 6) to highlight the spatial locations with signal changes that were considered most relevant by the DL pipeline for predicting the associated Behavior_SYL_.

The same DL pipeline was also trained using only the first hour of data per animal, except that (1) input data were not balanced; (2) in M3, two additional ‘R’ blocks were added from outside the 1-h sample to ensure that the ‘R’ class met the minimum data size requirement; and (3) performance was evaluated on the remaining 23 h of data.

#### Sanity check no. 1

As outlined previously^51^, we randomly shuffled the ground truth labels of the Neu_ACT_ blocks, which destroyed the original correspondence between the information contained in Neu_ACT_ blocks and their true classes or Behavior_SYL_ (R, INT and MM). ResNet50 was then re-initialized from the same pretrained ImageNet weights and the ResNet50-BiLSTM model was retrained on datasets with shuffled labels. Subsequently, we compared the performance between ResNet50-BiLSTM models trained on three, independently randomized versions of neurosurveillance data versus that of a ResNet50-BiLSTM model trained on the original neurosurveillance dataset (Extended Data Fig. 5b–m). Data from mouse M2 was used for this comparison.

#### Sanity check no. 2

As outlined previously^52^, we hypothesized that if the model indeed learned to leverage temporal dynamics (not just spatial appearance or artifacts) for classification, then destroying temporal continuity in the input data should degrade its performance. To test this hypothesis, we randomly shuffled the relative position in time (the temporal location), of each Neu_ACT_ image within the 24-h neurosurveillance dataset from mouse M2. Random shuffling was performed twice, to generate two temporally incoherent datasets. Next, 1-min Neu_ACT_ sequences (Neu_ACT_ blocks), were reconstructed from the shuffled data. This procedure preserved all other image information, including the spatial distribution of Neu_ACT_ within each image while suppressing any temporal cues which the BiLSTM model could leverage.

### Computing microscopic-scale maps of seed-based functional connectivity

Seed-based functional connectivity maps were computed using ΔF/F data as described elsewhere^53^. A 5 × 5-pixel mean filter was used before functional connectivity computations.

### Parameterizing the brain tumor microenvironment

24-h periods from TI/TA phases were selected and motion-corrected via image registration with ImageJ (‘MultiStackReg’ and ‘TurboReg’ plugins^44^) or MATLAB (‘NoRMCorre’^54^) scripts. Next, mean images were computed over 5-min intervals and images from the TA phase co-registered to those the TI phase. Then, image stacks from both phases were cropped to retain only those portions of the FoV that were common to the images acquired from both phases. The TI and aggregation BTMEs were characterized by computing the following parameters for each image on a ~100 × 100-μm (16 × 16-pixel) grid: (1) fractional occupancy of brain tumor cells (FA_C_); (2) fractional occupancy by blood vessels (FA_V_); (3) MVD; (4) MVL; (5) Tort; (6) AVG_CBF_; and (7) CoV_CBF_.

### Reporting summary

Further information on research design is available in the Nature Portfolio Reporting Summary linked to this article.

## Online content

Any methods, additional references, Nature Portfolio reporting summaries, source data, extended data, supplementary information, acknowledgements, peer review information; details of author contributions and competing interests; and statements of data and code availability are available at 10.1038/s41592-026-03111-z.

## Supplementary information

Supplementary Information Supplementary Tables 1–6, Supplementary Figs. 1–14 and Supplementary Note 1.

Reporting Summary

Supplementary Video 1 High-throughput neurosurveillance in multiple animals using a ‘bank’ of CloudScopes.

Supplementary Video 2 Video showing the three murine behavioral syllables: MM, INT and R, while simultaneously conducting neurosurveillance via a CloudScope. The video has been sped up by 10×.

Supplementary Video 3 A montage of videos from five animals bearing the CloudScope to illustrate that the ultralight and thin flexible PCB cable did not impede their free/natural behavior over 24 h. Time stamps during the video indicate images from 1 h, 12 h and the 24 h of continuous neurosurveillance.

Supplementary Video 4 High-speed image acquisition with the CloudScope’s livestream mode. **a**, Characterizing the vascular transit of an intravenously injected fluorescence tracer (that is FITC-Dextran) with high-resolution (512 × 512-pixel images) at ~9 frames per second. **b**, Corresponding arrival times, shown with regard to the earliest arrival time. These tracer kinetic data were employed to discriminate arteries from veins. LSB, least significant bit.

Supplementary Video 5 Interrogating Neu_ACT_ (that is, Δ*F*/*F*) with high temporal resolution (original acquisition was at ~ 19 fps, resampled to 50 ms, 128 × 128-pixel images) over 10 min.

Supplementary Video 6 24-h raw videos. **a**–**d**, GCaMP6s-bound Ca^2+^ FL; IOS (**a**) under 530 nm ± 15 nm (**b**) and 680 nm ± 1 nm illumination (**c**); and LSC-based CBF, for a representative animal (**d**). Videos are pseudocolored using green (black, low; green, high, for FL), grayscale (black, low; white, high, for IOS) and jet (blue, low; red, high, for LSC) colormaps, respectively. Videos are sped up to show 1 min of data per second. Time stamps are included in all video frames.

Supplementary Video 7 ‘Neurosurveillance dashboard’ for visualizing 24-h behavioral and neurosurveillance data. Data are shown for animal M2. For visualization purposes, noise in CBF maps was suppressed using equation (3) and that in Hb_SAT_ images was suppressed by applying a nonlocal means average filter (neighborhood size = 300 × 300 μm, respectively).

Supplementary Video 8 **a**–**e**, Videos illustrating the overlap (yellow) of pseudocolored maps of peak (top 50%) Neu_ACT_ (blue) and peak CBF (red) computed every 15 min over 24 h in animals M1–M5, respectively.

Supplementary Video 9 **a**–**d**, Video snippets showing: Neu_ACT_, ΔHbT, CBF and Hb_SAT_ changes during a PTZ-induced seizures (SZ_D_) in animals, M1–M4, respectively. Time stamps are included every 5 s with *t* = 0 s being the time point at which the seizure exhibited peak Neu_ACT_. For visualization purposes, noise in CBF maps was suppressed using equation (3) and that in Hb_SAT_ images was suppressed by applying a nonlocal means average filter (neighborhood size of 300 × 300 μm).

Supplementary Video 10 **a**–**c**, Video snippets showing: Neu_ACT_, ΔHbT, CBF and Hb_SAT_ changes during spontaneous seizures (SZ_SP_) in animals, M1 (*n* = 1), and M2 (*n* = 2), respectively. Time stamps are included every 5 s with *t* = 0 s being the time point at which the seizure exhibited peak Neu_ACT_.

Supplementary Video 11 Neurosurveillance dashboard for visualizing 24-h behavioral and neurosurveillance data. Data are shown for 24 h from animal M2, during which it exhibited a drug-induced seizure and two spontaneous seizures. For visualization purposes, noise in CBF maps was suppressed using equation (3) and that in Hb_SAT_ images was suppressed by applying a nonlocal means average filter (neighborhood size of 300 × 300 μm).

Supplementary Video 12 **a**, Migration trajectories for 100 GL261–GFP brain tumor cells that were tracked over 72 h over a wide FoV via neurosurveillance with the CloudScope. **b**–**d**, Unique regions of interest (ROI) from the same field illustrating: two glioma cells merging (**b**); glioma cells co-opting and intravasating into a blood vessel (**c**); and a glioma cell migrating to a distant location (displacement >100 µm) (**d**). Data are shown for animal M6.

Supplementary Video 13 **a**,**b**, 24-h videos showing a unique ROI during the TI phase in which glioma cells can be seen co-opting pre-existing blood vessels (purple, blood vessels; green, tumor cells) (**a**) and the co-opted vasculature exhibiting an attenuation in CBF (**b**). Data are shown for animal M6.

Supplementary Video 14 **a**–**c**, Video showing glioma cell dynamics and growth (**a**); vascular alterations (**b**); and perfusion changes (**c**) that occur within a FoV over 24 h, during the TI phase. Data are shown for animal M6. For visualization purposes, noise in CBF maps was suppressed using equation (3).

Supplementary Video 15 **a**–**c**, Video showing glioma growth (**a**); vascular alterations (**b**); and perfusion changes (**c**) that occur within a FoV over 24 h, during the TA phase. Data are shown for mouse M6. Noise in CBF images was suppressed by using the relative distribution of CBF in a reference CBF map (neighborhood size of 100 × 100 μm).

Supplementary Video 16 Spatial maps summarizing microscopic (cellular: FA_cell_); mesoscopic (vascular morphology: F_V_, MVD, MVL and Tort); and macroscopic (functional, AVG_CBF_ and CoV_CBF_) changes characteristic of the TI and TA phases. FA_cell_, tumor cell fractional occupancy; F_V_, vascular fractional occupancy; MVD, mean vessel density; MVL, mean vessel length; Tort, vascular tortuosity; AVG_CBF_, average CBF; CoV_CBF_, temporal coefficient of variation of CBF.

Supplementary Video 17 **a**–**e**, Maps of CBF computed from 100 successive raw laser speckle images using spatial neighborhoods of 3 × 3 (**a**), 5 × 5 (**b**), 11 × 11 (**c**), 51 × 51 (**d**) and 101 × 101 pixels (**e**), respectively.

## Data Availability

The 24-h neuroimaging data from a representative animal are available via the CodeOcean repository at 10.24433/CO.4182092.v1. Data are provided as 24-h time-series of 64 × 64-pixel images for each contrast channel. STL files for 3D printing CloudScope parts are also included in the same repository.

## Code availability

Image-processing scripts central to the findings of this study, for example pre-processing of images and visualizations for Figs. 1 and 3 are included in the CodeOcean repository at 10.24433/CO.4182092.v1. Output data were manually uploaded and included for processing steps that were not executable within the CodeOcean repository due to its resource constraints. Code reuse is permitted under the GNU General Public License v.3 (https://opensource.org/license/gpl-3.0).
