# Supplementary material for: A cloud-based miniscope for neurosurveillance of brain health and disease in freely behaving animals
Source: Nat Methods. 2026 Jun 22;23(7):1424–36. doi: 10.1038/s41592-026-03111-z (PMC13345946; doi:10.1038/s41592-026-03111-z)
Supplement: Supplementary file 1 — Supplementary Tables 1–6, Supplementary Figs. 1–14 and Supplementary Note 1. [file 41592_2026_3111_MOESM1_ESM.pdf]

nature methods

Supplementary information https://doi.org/10.1038/s41592-026-03111-z

A cloud-based miniscope for neurosurveillance of brain health and disease in freely behaving animals

In the format provided by the authors and unedited

SUPPLEMENTARY TABLES

CNS disease model	Selected neuropathologies of interest	Corresponding time scales
Seizures	(i) Occurrence of spontaneous seizures	Seconds-minutes[1], spread over hours or days[2]
	(ii) Neurologic recovery from seizures	~1 hour or more[3]
Brain tumors	(i) Tumor cell migration dynamics	Hours/days at speeds = 5-100 µm/hour[4,5]
	(ii) Vascular co-option	Hours[6]/days[4]
	(iii) Angiogenesis	Days[7]
Stroke	(i) Hypoxic ischemic injury	Seconds-hours[8,9]
	(ii) Recovery	Days/weeks[10]
Brain injury from cardiac arrest and resuscitation	(i) Ischemic insult	Minutes[11]
	(ii) Reperfusion injury	~30 minutes[12]
	(iii) Post-resuscitation hypoperfusion	>hours[13]
Traumatic brain injury	(i) Post-insult thrombogenesis	Minutes-hours[14]
	(ii) Progressive axonal damage	Days[15]
Migraine	(i) Pattern of cortical spreading depressions	Seconds-minutes[16]
Alzheimer's disease	(i) Altered ultraslow fluctuations	Seconds-minutes[17]
	(ii) Blood pressure/flow anomalies	Hours/days or more[18]
Schizophrenia	(i) Disrupted circadian rhythms	Hours/days[19]

Supplementary Table 1

Report	Weight (g)	Imaging contrast mechanism			Spatial resolution (µm)	Field of view (mm)	Frame rate (Hz)	Duration of continuous imaging in freely behaving animals
		FL	IOS	LSC				
Ghosh et al, 2011[20]	1.9	×			2.5-2.8 ***	0.6×0.8	36-100	45 mins
Miao et al, 2011[21]	20			×	62.5 ***	12.8×14.5	50	20 mins
Liu et al, 2013[22]	1.5		×	×	5.5 **	2.6×3.5	2-50	10 mins
Cai et al, 2016 (UCLA Miniscope V3)[23]	3	×				0.7×0.45	30-60	10 mins
Liberti et al, 2017 (FinchScope)[24]	1.8 (wl)<br>3.8 (wired)	×			4.4 ***	0.8×0.6	30	60 mins (wl)
Jacob et al, 2018 (CHEndoscope)[25]	4.5	×				0.5×0.5	20	
Skocek et al, 2018 (MiniLFM)[26]	2.7-4	×			12.4 ***	0.7×0.7×0.36	16	1 hour
Barbera et al, 2019[27]	3.9 +<br>3.8 (bat)	×			5 *	0.5×0.5	10	40 mins
Yanny et al, 2020[28]	2.5	×			5.5-7.8 ***	0.9×0.7×0.4	40	
Shuman et al, 2020 (UCLA Miniscope V4, wireless version)[29]	4-5	×			1.7 ***	0.55×0.55	20	45 mins
Juneau et al, 2020 (MiniFAST)[30]	3.45	×			0.8 ***	5.5×3.1	30	2 hours
de Groot et al, 2020 (NiNScope)[31]	1.6	×			1.0 *	0.8×0.6	30	2 hours
Scherrer et al, 2021 (FeatherScope and KiloScope)[32]	1.0 and 1.4	×			2.2 (center) ***	1.0×1.0<br>and<br>4.8×3.6		Minutes
Rynes et al, 2021[33]	3.8	×	×		35.1 **	8.0×10.0	15	2 hours
Zong et al, 2021 (FHIRM-TPM)[34]	2.4-4.2	×			1.1 (lat) ††<br>12.2 (ax) ††	0.4×0.4×0.2	20	7 mins
Zong et al, 2022 (Mini2P)[35]	2.4	×			1.2 (lat) †††<br>12.8-17.8 (ax) †††	0.5×0.5	15-40	1 hour
Supekar et al, 2022 (SIMscope3D)[36]	6.7	×			1.4 (lat) †††<br>18.1 (ax) †††	0.2×0.2×0.55	3.5 min/stack	1 hour
Zhao et al, 2023[37]	2.2	×			1.0 (lat) ††<br>7.2 (ax) ††	0.4×0.4×1.0	16	2 hours
Guo et al, 2023[38]	13.9	×			2.1-2.2 †††	3.6×2.7	100	15 mins
Zhang et al, 2024[39]	2.5	×			4.0 †††	3.6×3.6	16	10 mins or more
Madruga et al, 2024 (UCLA 2P Miniscope)[40]	4	×			1.0 (lat) †<br>10.2 (ax) †	0.45×0.38	-	60 mins
Dong et al, 2024[41]	4.8	××			1.8 **	1.0×1.0	20	15 mins
Senarathna et al, 2019 (our prior work)[42]	9 (or 3 w. relief)	×	×	×	5.0 *	3.0×3.0	1× contrast: 15<br>3× contrasts: 1 min/cycle	1 hour
**Current work**	**~3.5**	**×**	**×**	**×**	**≥ 7.0 **×**	**3.0×3.0**	**1× contrast= up to ~19<br>3× contrasts= ~0.2/contrast**	**1× contrast: 10 mins<br>3× contrasts:>24 hours**

Supplementary Table 2

Imaging modality	Image Size (in pixels)	Exposure time (ms)	Analog gain	Comments
FL	512 × 512	50 - 200	4 - 6	Maximize exposure time and analog gain to improve SNR
IOS	512 × 512	50 - 200	1 - 6	Set exposure time and gain to minimize glares induced by specular reflections
LSC	1536 × 1536	20 - 50	1	Increase pixel-count and minimize analog gain to improve SNR of laser speckle contrast

Supplementary Table 3

Animal ID	Run (Randomly selected samples)	Intermediary (Randomly selected samples)	Minimally mobile (Randomly selected samples)	The ratio of the original dataset (Approx.)
M1	116 (all)	144 (all)	911 (150)	2:03:18
M2	43 (all)	441 (100)	812 (100)	1:10:20
M3	21 (all)	312 (50)	1004 (50)	1:15:50
M4	73 (all)	417 (100)	784 (100)	1:06:10
M5	52 (all)	340 (100)	903 (100)	1:07:18

Supplementary Table 4

Mouse ID	Batch size	Dropout	Decay rate	Learning rate	Patience (Learning rate scheduler)	Early stopping (iterations)	Maximum number of iterations	Train-validation - test split (%)
M1	32	0.295	0.279	0.0001325	3	14	100	69-21-10
M2	32	0.122	0.527	0.0003900	12	30	100	74-16-10
M3	32	0.326	0.275	0.0015720	4	21	100	69-21-10
M4	32	0.497	0.836	0.0018053	14	16	100	69-21-10
M5	32	0.213	0.708	0.0003964	10	21	100	69-21-10

Supplementary Table 5

Mouse ID	Macro-averaged F1 scores across 5-folds
M1	83%±5%
M2	89%±9%
M3	88%±13%
M4	81%±6%
M5	80%±4%

Supplementary Table 6

SUPPLEMENTARY FIGURES

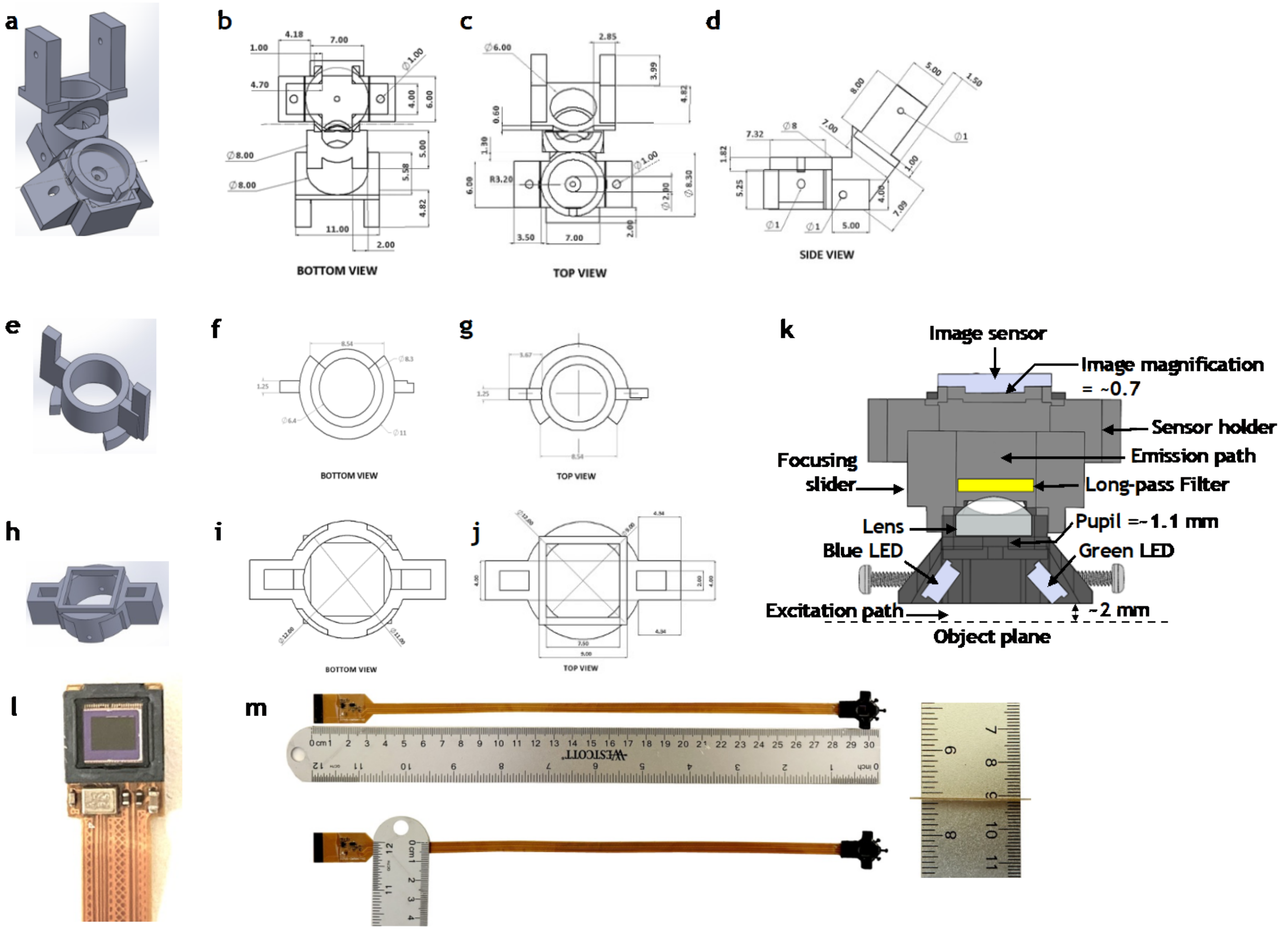

Supplementary Fig. 1: Additional CloudScope design specifications. (**a**) 3D rendering of the CloudScope base and schematic views of the: (**b**) bottom, (**c**) top, and (**d**) side. (**e**) 3D rendering of the focusing slider and schematic views of the: (**f**) bottom and (**g**) top. (**h**) 3D rendering of the image sensor holder and schematic views of the: (**i**) bottom and (**j**) top. (**k**) Cross-section of CloudScope showing fluorescence excitation and emission light paths. (**l**) Top view of the image sensor, and (**m**) views of the flexible and ultrathin image sensor cable showing the flexible PCB-based image sensor controller, alongside a ruler for scale. Note: In some instances, cyanoacrylate glue was used to secure 3D printed components (e.g. the sensor holder to the focus slider, or the focus slider to the miniscope base) to maintain a tight fit. The schematic in (**k**) was created using SolidWorks 2021 (Dassault Systèmes, France).

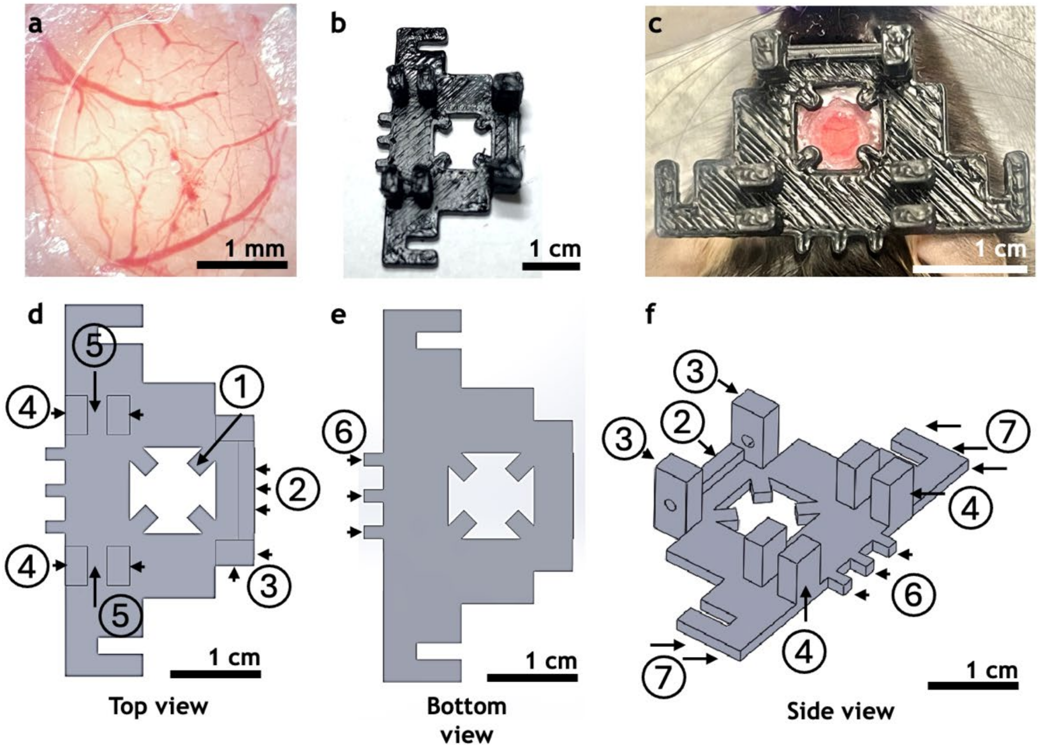

Supplementary Fig. 2: 3D-printed head-mount for attaching the CloudScope. (**a**) View of the mouse cortex through a cranial window under white light illumination. (**b**) Photograph showing a 3D printed head-mount for attaching the CloudScope to the mouse's skull. The head-mount weighs ~0.5 g and is positioned on the exposed skull such that the central opening is directly over the cranial window. (**c**) Photograph showing the head-mount over a cranial window. Schematics showing the (**d**) top, (**e**) bottom, and (**f**) side view of the head-mount with circles indicating key features. These include: (1) prongs to guide head-mount placement. The head-mount is placed such that the intersection of the four prongs lies at the center of the cranial window (as shown in **c**); (2-4) scaffolds that immobilize the CloudScope once it has been inserted into the head-mount; (5) slots for screws to attach the CloudScope to the head-mount; (6) additional prongs for gluing the head-mount to the skull. The roughness of the head-mount's bottom surface aids adhesion. (7) Side bars assist in manipulating the head-mount. Once the head-mount is attached to the skull, these side bars are used to keep the animal's head still when focusing the CloudScope. The schematics in **d-f** were created using SolidWorks 2021 (Dassault Systèmes, France).

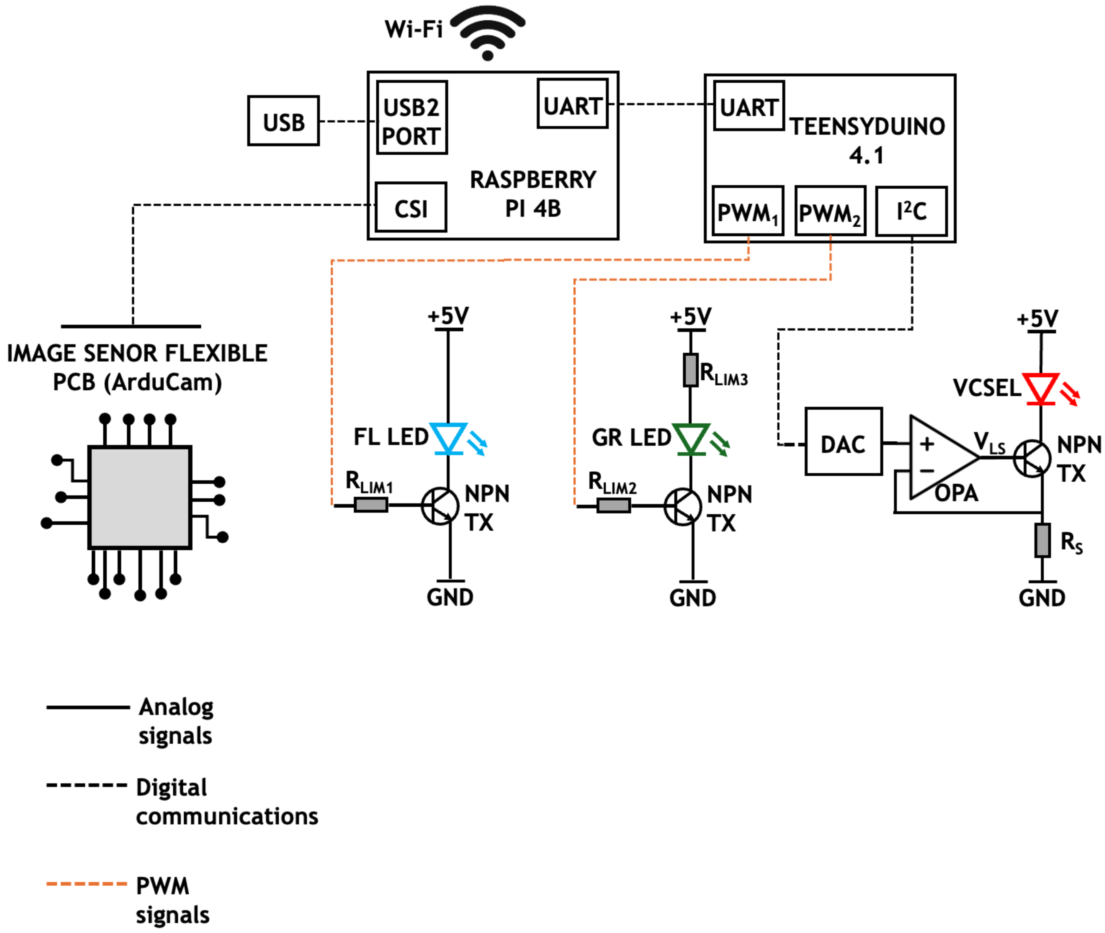

Supplementary Fig. 3: CloudScope's control electronics. The CloudScope is controlled by a Raspberry Pi 4B processor and a Teensyduino 4.1 microcontroller unit. The Raspberry Pi handles all communications with the cloud-based server by connecting to the internet via local Wi-Fi. It also stores acquired images on a USB drive attached to one of its USB 2.0 (i.e., USB2) ports. In addition, the Raspberry Pi communicates via its camera serial interface (CSI) with ArduCam's flexible PCB that controls an OV5647 image sensor. All commands to set image sensor-related parameters (e.g. exposure time, analog and digital gains etc.) and image data are transferred via this interface. Control of illumination parameters is delegated to the Teensyduino 4.1. The two modules are in an "initiator-responder" configuration wherein the Teensyduino listens to commands from the Raspberry Pi via its UART port. Once information specifying an illumination source and its brightness level is received, the Teensyduino uses one of its pulse width modulation (PWM) modules (i.e. PMW1 and PMW2 modules for BL and GR illumination) or its I2C module (i.e. for LS illumination) to switch ON/OFF the appropriate illumination source. The PMW modules use a standard load bearing circuit with an NPN transistor and current limiting resistors (e.g. R_{LIM}s) to drive the BL and GR LEDs. In contrast, the I2C module communicates with a 12-bit digital to analog converter (MCP4725) to create an analog voltage (V_{LS}). V_{LS} is converted to the appropriate current level to drive the laser diode (i.e. the VCSEL) via an operational amplifier (OPA, TLV2462) based constant current circuit. The resistor R_s is used to set the proportionality constant for the voltage to current conversion. Analog signals, digital communications, and PWM signals are shown via solid black, dashed black, and dashed orange lines, respectively. The image sensor symbol was created with Biorender.com.

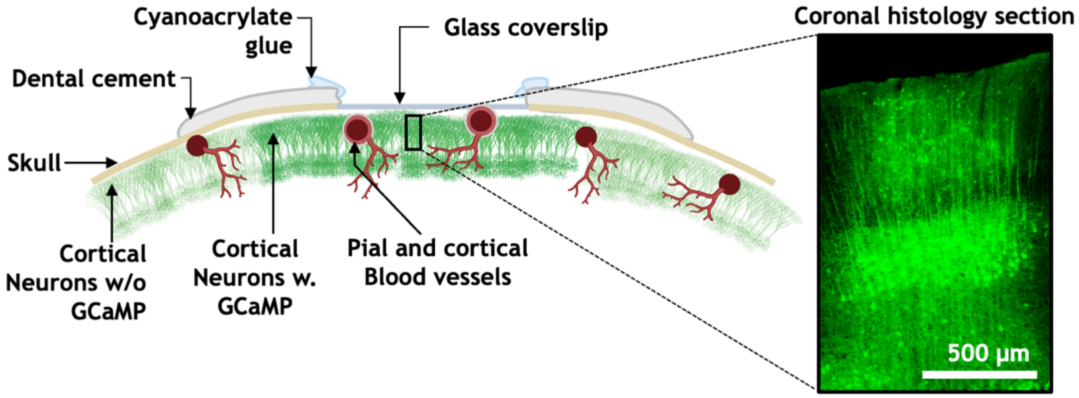

Supplementary Fig. 4: Cranial window preparation for healthy/seizure experiments. Schematic showing a cross-sectional view of the cranial window used for neurosurveillance in healthy and seizure animals. The window was centered on +2/-2 mm ML/AP and consisted of a 100 µm-thick 3 mm-diameter glass cover slip. A combination of cyanoacrylate glue and dental cement was used to secure the cover slip. Cortical neurons under the cranial window were virally transfected to express GCaMP, and its expression was verified via ex vivo two-photon imaging of coronal brain sections as shown in the zoomed inset. Cortical neurons outside the cranial window were not virally transfected with GCaMP and did not express GCaMP. Also shown in the schematic are representative pial and cortical microvessels located under the cranial window. The schematic was created with Biorender.com.

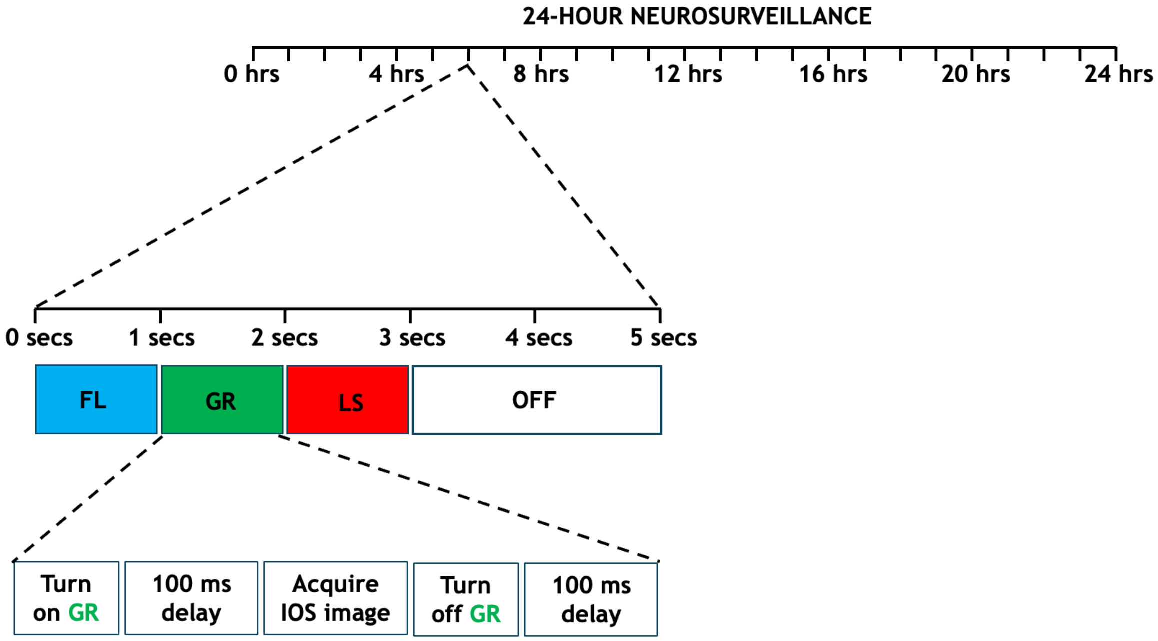

Supplementary Fig. 5: Timing diagram for sequential image acquisition. 24-hour neurosurveillance was conducted by cycling through the FL, IOS, and LSC channels every ~5 s. For image acquisition under each channel, the appropriate illumination source was first switched on at a predetermined intensity, i.e. the blue LED (BL) for FL, green LED (GR) for IOS, and the laser diode (LS) for LSC. Next, appropriate changes to image acquisition parameters, i.e. exposure time, analog gain, and image size, were made. Following this, the Pi's image buffer was cleared to discard old images, and a single image acquired and saved. Finally, the illumination source was switched off. Although images were typically acquired with exposure times ranging from 50-200 ms, steps such as illumination control, changes to acquisition parameters, and resetting the image buffer that were implemented via object-oriented programming with open-source software could add ~600-800 ms, resulting in ~1 s per imaging modality. Following image acquisition for all three channels, the image sensor was briefly (i.e. ~2 s) switched off to prevent overheating. For brain tumor imaging, each image acquisition cycle was padded with an additional 25 s of off time, resulting in timing of ~30 s per cycle. **NOTE:** the duration of each cycle, and of the steps within each cycle varied because of hardware/software bottlenecks inherent to the Raspberry Pi. Therefore, millisecond-resolution timestamps were incorporated into each image file's name. Accessing these time stamps offline permitted resampling image time series with 5-second temporal resolution prior to all analyses.

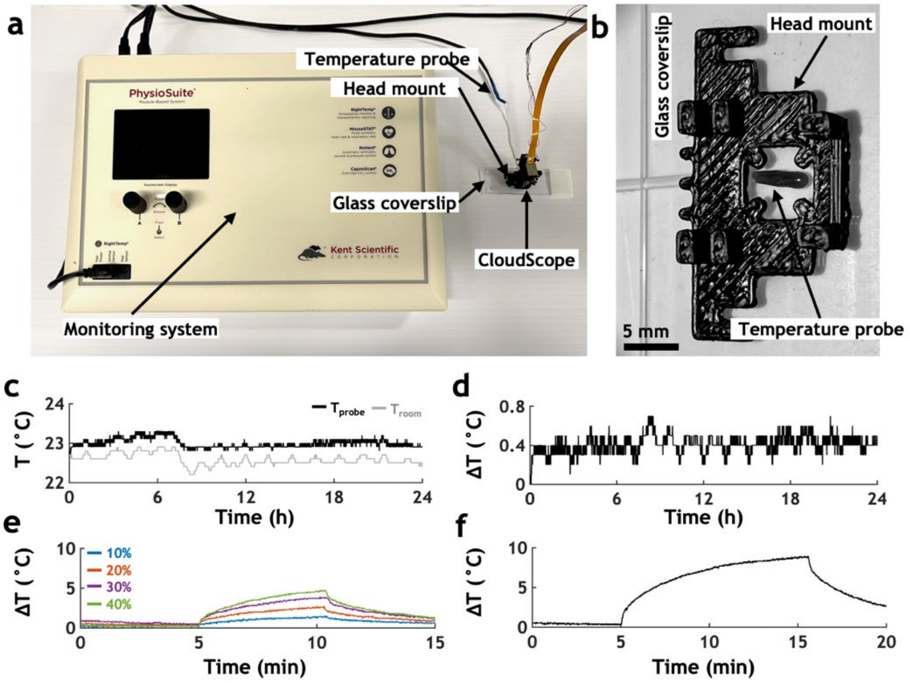

Supplementary Fig. 6: **Phantom-based temperature measurements of CloudScope operation**. (**a**) Phantom setup for temperature measurement during CloudScope operation. Temperature was a mouse temperature probe placed below a 1-mm thick glass coverslip atop which a head-mount was affixed. (**b**) The probe's temperature was recorded using a Kent Scientific Physiosuite monitoring system during CloudScope operation. Room temperature was monitored via an external temperature monitor placed besides the setup. (**c**) Timeseries of room (grey, i.e. T_{RM}) and probe (black, T_{PR}) temperature during a 24-hour-long imaging session conducted under CloudScope's 'sequential' mode. Imaging was run using the same set of acquisition and illumination parameters used for in vivo neurosurveillance. Values are plotted every 30 s. (**d**) Time series of temperature change due to CloudScope operation, i.e., $\Delta T = T_{PR} - T_{RM}$. (**e**) Time series of temperature changes arising from operating CloudScope in the 'live stream' mode for 5 minutes. For comparison, pre- and post-operation time series are shown over additional 5-minute durations. Time series are shown for fluorescence illumination intensities of 10%, 20%, 30%, and 40%, which are typically used for acquiring FITC-Dextran kinetics. These settings raised the phantom temperature by 0.69±0.40 °C, 1.24±0.73 °C, 1.84±1.08 °C, and 1.98±1.51 °C, respectively.
(**f**) The time series of temperature changes arising from 'live stream operation' for a longer-duration with the settings used for acquiring the high-framerate dataset shown in **Extended Data Fig. 2** (i.e. 10 minutes, 65% fluorescence illumination intensity). Our recorded temperature increase of the phantom (i.e. 4.68±3.05 °C) was similar to that estimated for standard two-photon *in vivo* imaging experiments [43]. 5 minute-long pre- and post-operation periods are also shown.

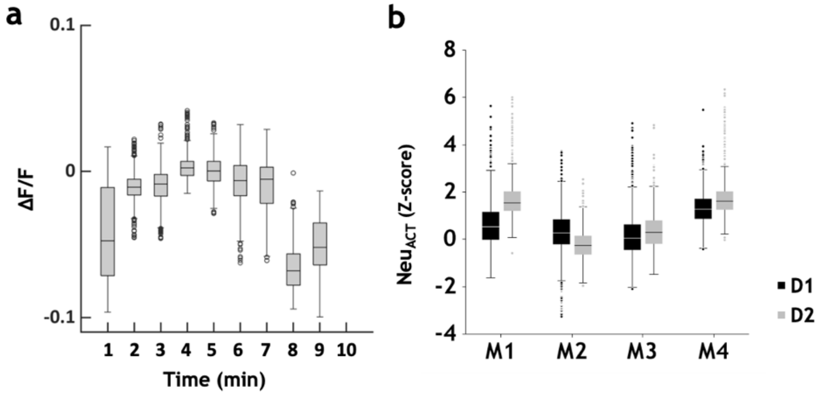

Supplementary Fig. 7: **Photobleaching was not observed during CloudScope operation**. (**a**) The distribution of Δ**F/F** corresponding to the behavioral syllable MM, observed during each 1-minute period over 10-minutes of high-speed image acquisition in M4. The lack of a clear trend suggests that brain activity was not substantially altered. (**b**) The distributions of Neu_{ACT} in mice M1-M4 corresponding to the behavioral syllable MM during the first 2 hours of 24-hour neurosurveillance. For each mouse, data are shown for the initial (i.e. healthy or D1) and subsequent (i.e. day of the seizure or D2) imaging sessions (seizure-related changes were excluded). The lack of a clear decreasing trend from D1 to D2 indicates minimal photobleaching. In both boxplots, the lower bound, center, and upper bound of the boxes indicate the 1st, 2nd, and 3rd quartiles. The top and bottom whiskers connect the 3rd and 1st quartiles to the maximum and minimum values considered not to be outliers, respectively. The maxima and minima for non-outliers were defined as those within 1.5× of the inter-quartile range, i.e. the difference between the first and the third quartile, from the box edges.

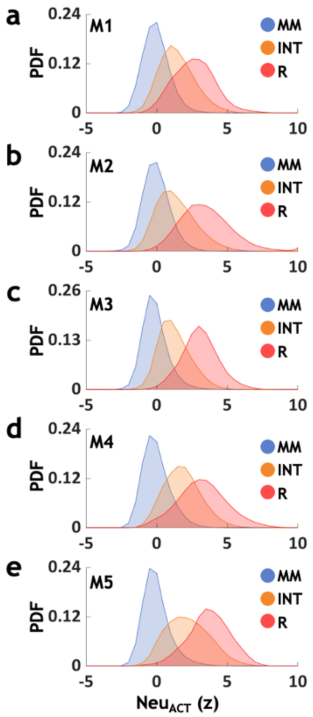

Supplementary Fig. 8: **Behavior vs neuronal activity**. (**a**-**e**) Distributions of parenchymal Neu$_{ACT}$ corresponding to behavioral syllables MM, INT, and R over 24-hours, in animals M1-M5, respectively.

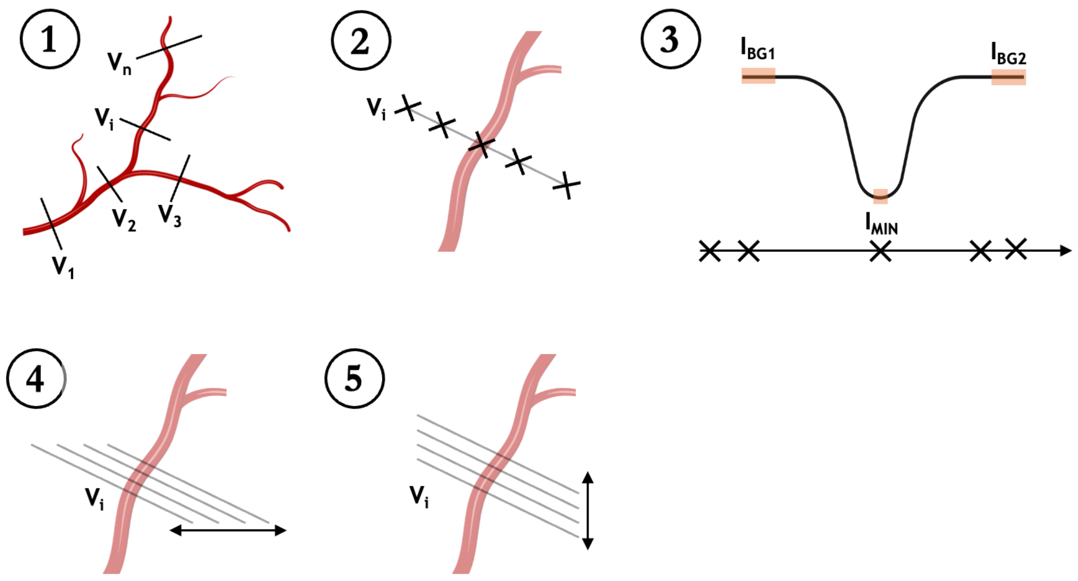

Supplementary Fig. 9: Algorithm for characterizing vasomotor dynamics. Step 1: Line segments (e.g. V_1, V_2, V_3, …, V_i, …, V_n) were manually selected across each vessel segment. Care was taken to include a reasonable number of pixels. (e.g., >10) on either side beyond the vessel diameter. **Step 2**: For each line segment, e.g. V_i, intensity values (I) recorded under isosbestic wavelength (e.g. 530 nm or 570 nm) illumination were extracted. **Step 3**: These intensities were plotted against their Euclidian distance with respect to the starting coordinate of that line segment. Next, mean intensity values for background regions from either side of the vessel (i.e. I_{BG1} and I_{BG2}) and the minimum intensity value (I_{MIN}) were extracted. As an added precaution, I_{MIN} was replaced by the mean intensity of that pixel and its two neighbors. These values were input to a novel vascular-centric approach for quantifying the vessel diameter (see **Methods**). **Steps 4, 5**: For resilience against motion, additional I_{MIN}, I_{BG1}, and I_{BG2} were computed by jittering the line segment horizontally and vertically and then input to the same algorithm. The mean diameter resulting from every jitter location was used as the vessel diameter.

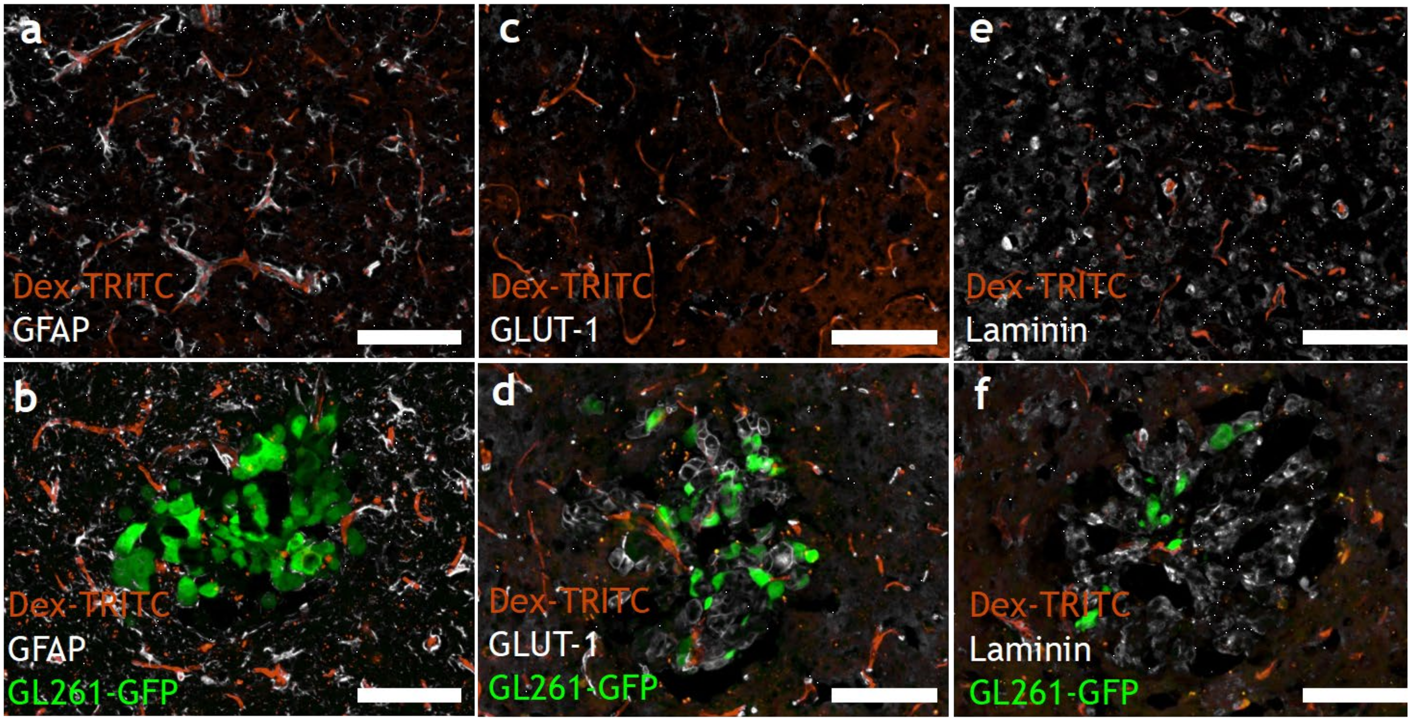

Supplementary Fig. 10: Immunohistochemical (IHC) validation of the BTME phenotype: (**a**) Non-glioma field-of-view (FOV) from the glioma-bearing animal that underwent in vivo imaging in which astrocytes were labeled with anti-GFAP (IR channel) and blood vessels with dextran-TRITC (red channel). The intimate coverage of the cortical vessels with astrocytes is evident. In contrast, (**b**) the GL261-GFP glioma (green channel) bearing FOV illustrates blood vessels with dissociated GFAP or lacking GFAP coverage. Additionally, GFP-expressing tumor cells can be seen co-opting the cortical vasculature. (**c**) Non-glioma FOV in which the blood-brain-barrier (BBB) was labeled with anti-GLUT-1 (IR channel) and blood vessels with dextran-TRITC (red channel). The intact BBB of cortical vessels is evident. In contrast, (**d**) the GL261-GFP glioma (green channel) bearing FOV exhibits some blood vessels with limited BBB labeling as well as extensive GLUT-1 labeling in and around brain tumor cells. (**e**) Non-glioma FOV in which the vascular basement membrane was labeled with anti-laminin (IR channel) and blood vessels with dextran-TRITC (red channel). The intact basement membrane of cortical vessels is evident. In contrast, (**f**) the GL261-GFP glioma (green channel) bearing FOV exhibited blood vessels with limited or no laminin labeling, as well as extensive laminin labeling in and around brain tumor cells. Collectively, these IHC data validate the phenotype of the BTME as being one that exhibits co-optive brain tumor growth, aberrant cortical vasculature, and a remodeled perivascular niche. All images were acquired at 20×. Scale bar = 100 µm.

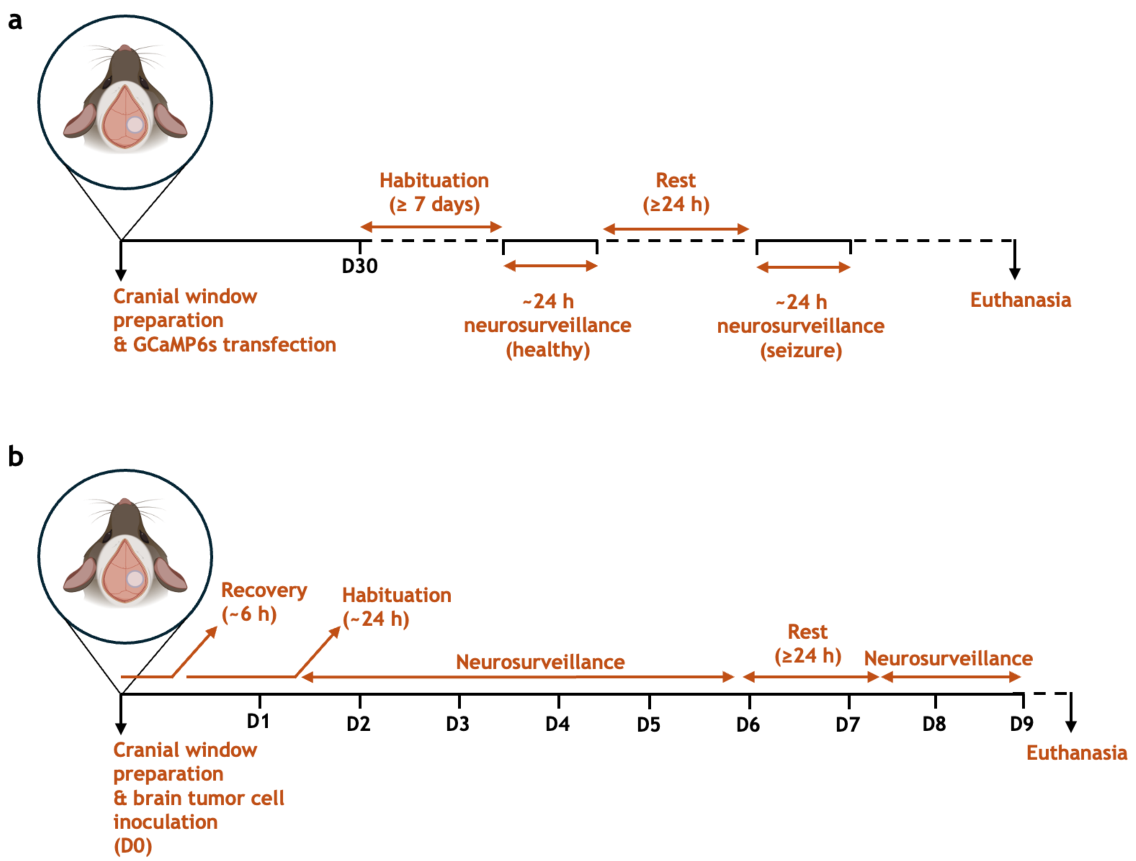

Supplementary Fig. 11: Experimental timelines. (**a**) The timeline for healthy and seizure neurosurveillance. FITC-dextran injections were performed under anesthesia, either prior to neurosurveillance (M2), after the first 24-hour imaging session (M3 and M5), or after the second 24-hour imaging session (M1). Habituation comprised of housing mice in an open-top cage that permits CloudScope operation, and one or two ~24-hour-long sessions of wearing the CloudScope. (**b**) Timeline for neurosurveillance of the brain tumor microenvironment (BTME) in M6. Neurosurveillance during D1-D5 included intermittent breaks spanning a total of ~1 hour. In contrast, neurosurveillance of the BTME in M6 was only conducted for days ~D1-D5.

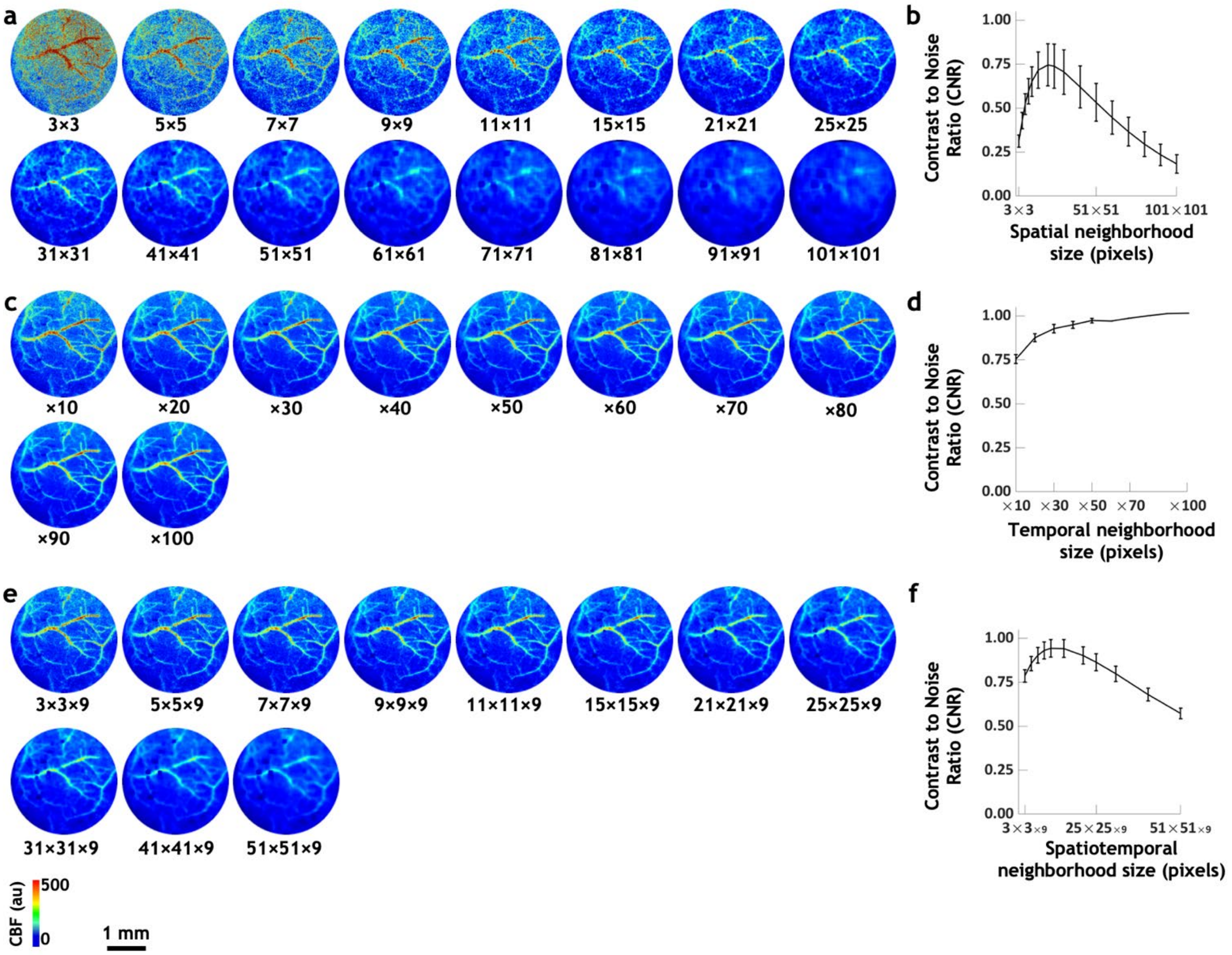

Supplementary Fig. 12: Effect of the spatial and temporal neighborhood size on computation of cerebral blood flow (CBF) maps. (**a**) Panel showing maps of CBF computed from a representative raw laser speckle image using spatial neighborhood sizes of 3×3, 5×5, 7×7, 9×9, 11×11, 15×15, 21×21, 25×25, and 31×31 pixels. All data are from mouse M3. (**b**) Graph showing the distribution (mean ± standard deviation) of contrast-to-noise ratios (CNR) for visualizing the microvasculature as a function of the spatial neighborhood size. CNR was computed as the ratio of the mean CBF differential between vessel and background regions against the standard deviation of CBF in background regions. A vessel mask created from IOS images was used to identify vessel vs background pixels. CNR distributions were computed over CBF maps created from n=100 representative raw speckle images. **Supplementary video 17** show time-lapse CBF maps (n=100) corresponding to each spatial neighborhood size used above. (**c**) Panel showing representative maps of CBF computed using temporal neighborhood sizes of 10, 20, 30, 40, 50, 60, 70, 80, 90, and 100 pixels. (**d**) Graph showing the distribution of CNR (mean ± standard deviation) as a function of temporal neighborhood size. CBF maps were computed using the same n=100 raw speckle images used in (**b**). Standard deviations are included for temporal neighborhood sizes of 10-50 pixels, as multiple CBF maps could be created from n=100 raw speckle images (e.g. 5 CBF maps could be created for a temporal neighborhood size of 20 pixels). (**e**) Panel showing representative maps computed using spatial neighborhoods of: 3×3×9, 5×5×9, 7×7×9, 9×9×9, 11×11×9, 15×15×9, 21×21×9, 25×25×9, 31×31×9, 41×41×9 and 51×51×9. (**f**) CNR distributions (mean ± standard deviation) as a function of spatial neighborhood size.

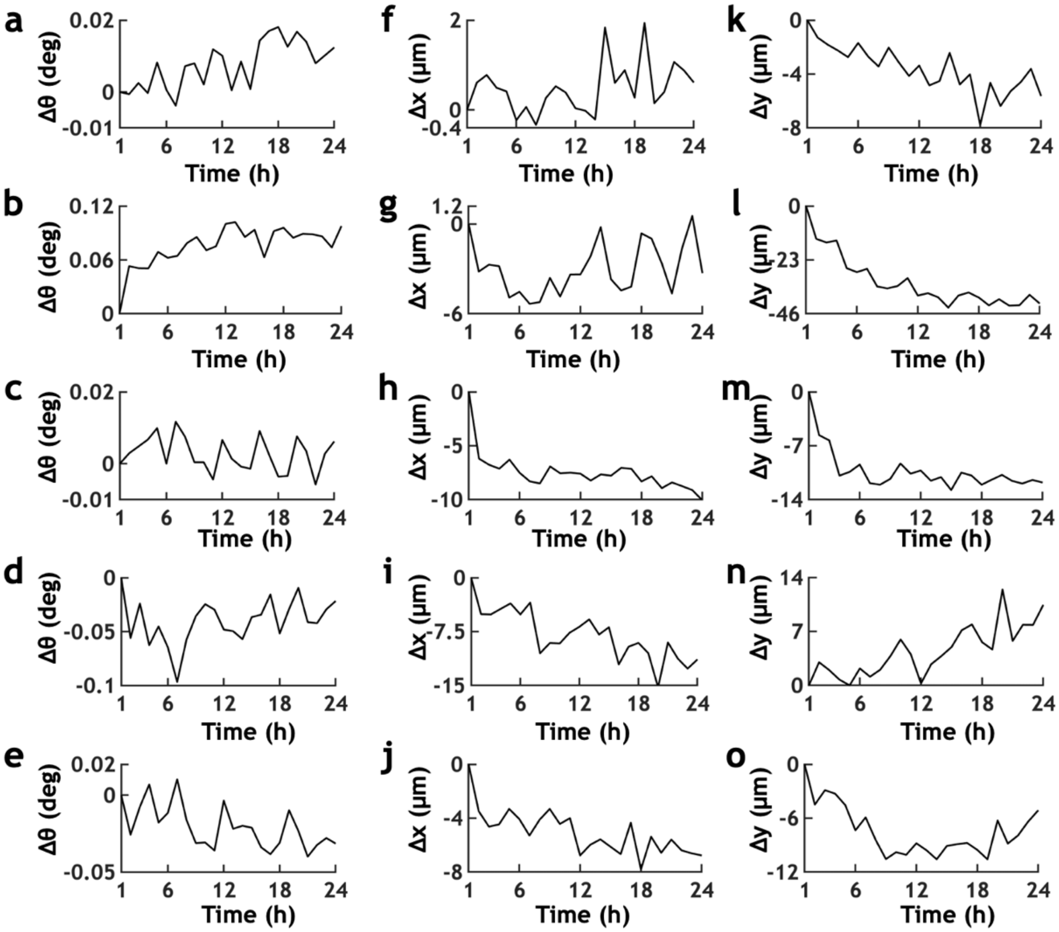

Supplementary Fig. 13: Motion artifacts encountered during neurosurveillance. (**a-e**) angular (i.e. ΔΘ) and (**f-j**) Δx and (**k-o**) motion artifacts encountered during 24 hours of neurosurveillance in mice M1-M5, respectively**.** Motion artifacts were estimated once per hour using the Analysis of Functional Neuroimaging (AFNI) software.

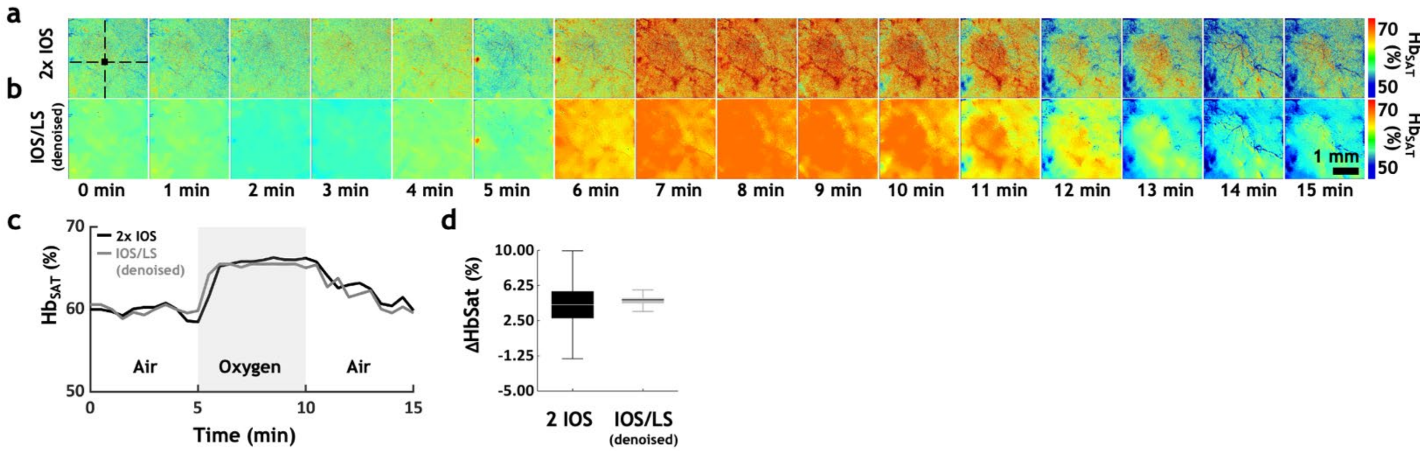

Supplementary Fig. 14: **Validation of the non-local means filter for suppressing speckle-induced noise in Hb_{SAT} maps**. (**a-b**) Montages showing time lapse images of Hb_{SAT} computed from using two IOS wavelengths (i.e. 530/600 ± 10 nm, **a**) and a combination of IOS/laser wavelengths (i.e. IOS=530±10 nm and laser=632.8 nm, **b**). A non-local means filter (kernel=51×51 pixels) was applied to the IOS/LS-based Hb_{SAT} maps. Images were acquired from the brain of an anesthetized mouse by using a benchtop-based imaging system. An oxygen inhalation challenge was performed from t=5-10 minutes. A 10×10-pixel region of interest (ROI) is marked. (**c**) Time series of Hb_{SAT} changes during inhalation challenge. (**d**) Box-plots indicating the distribution of mean Hb_{SAT} changes, i.e. ΔHb_{SAT} = μHb_{SAT} (Ox) - μHb_{SAT} (pre-Ox), computed from either technique. Here Ox=period of oxygen inhalation, i.e., 5-10 minutes, and pre-Ox=baseline prior to oxygen challenge, i.e. 0-5 mins. μ denotes the mean. ΔHb_{SAT} reported from the two-IOS wavelengths (4.2% ± 2.3%) were similar to those shown by the IOS/LS-combination (4.6% ± 0.6%). Furthermore, pixel-wise time series of Hb_{SAT} derived from each technique were tightly coupled, with a Pearson's correlation coefficient (i.e. R) of 0.71±0.15. Please also note that the non-local means filter was only used for visualization purposes. All data are from n=1 mouse. In the boxplot, the lower bound, center, and upper bound of the boxes indicate the 1st, 2nd, and 3rd quartiles. The top and bottom whiskers connect the 3rd and 1st quartiles to the maximum and minimum values considered not to be outliers, respectively. The maxima and minima for non-outliers were defined as those within 1.5× of the inter-quartile range, i.e. the difference between the first and the third quartile, from the box edges.

BEST PRACTICES FOR CLOUDSCOPE OPERATION

Additional considerations prior to using CloudScope: Since the CloudScope currently weighs 3.5 g, we recommend that it be used only to acquire images in adult mice (e.g. > 8 weeks). It is also necessary to sufficiently habituate the animal to the CloudScope prior to commencing neurosurveillance and securing the focus slider with cyanoacrylate glue prior to long-term (e.g. days) imaging. Furthermore, affixing the CloudScope at a large off-vertical angle should be avoided because it could create an undue torque on the mouse's head. Therefore, its use for imaging cortical regions substantially lateral to the midline (e.g. auditory cortex) should be undertaken with caution unless the animal's head is fixed[42].

Removing CloudScope following an experiment: Following the completion of each imaging experiment, mice were briefly anesthetized with isoflurane prior to removing the CloudScope. Since this maneuver can damage the wires powering the illumination sources, we gently draped them around the ribbon cable prior to introducing the animal into the anesthesia chamber.

Minimizing image sensor heating: To avoid overheating the image sensor, users are encouraged to limit continuous operation of the CloudScope's 'live stream' mode to ~10 minutes and allow a ~1-2 minutes break prior to the resumption of imaging. Continuous (i.e. >24 hours), 'sequential' mode operation requires a ~1.5-2.5 s break per ~5 s cycle.

REFERENCES

1 Zhao, M. *et al.* Preictal and ictal neurovascular and metabolic coupling surrounding a seizure focus. *J Neurosci* **31**, 13292-13300 (2011). https://doi.org/10.1523/JNEUROSCI.2597-11.2011

2 Durazzo, T. S. *et al.* Temporal distributions of seizure occurrence from various epileptogenic regions. *Neurology* **70**, 1265-1271 (2008). https://doi.org/10.1212/01.wnl.0000308938.84918.3f

3 Tran, C. H. T., George, A. G., Teskey, G. C. & Gordon, G. R. Seizures elevate gliovascular unit ca2+ and cause sustained vasoconstriction. *JCI Insight* **5**, e136469 (2020). https://doi.org/10.1172/jci.insight.136469

4 Winkler, F. *et al.* Imaging glioma cell invasion in vivo reveals mechanisms of dissemination and peritumoral angiogenesis. *Glia* **57**, 1306-1315 (2009). https://doi.org/10.1002/glia.20850

5 Farin, A. *et al.* Transplanted glioma cells migrate and proliferate on host brain vasculature: A dynamic analysis. *Glia* **53**, 799-808 (2006). https://doi.org/10.1002/glia.20334

6 Voutouri, C. *et al.* Experimental and computational analyses reveal dynamics of tumor vessel cooption and optimal treatment strategies. *Proc Natl Acad Sci U S A* **116**, 2662-2671 (2019). https://doi.org/10.1073/pnas.1818322116

7 Rege, A. *et al.* Longitudinal in vivo monitoring of rodent glioma models through thinned skull using laser speckle contrast imaging. *J Biomed Opt* **17**, 126017 (2012). https://doi.org/10.1117/1.JBO.17.12.126017

8 Yang, S. *et al.* Longitudinal in vivo intrinsic optical imaging of cortical blood perfusion and tissue damage in focal photothrombosis stroke model. *J Cereb Blood Flow Metab* **39**, 1381-1393 (2019). https://doi.org/10.1177/0271678X18762636

9 Sakadzic, S., Lee, J., Boas, D. A. & Ayata, C. High-resolution in vivo optical imaging of stroke injury and repair. *Brain Res* **1623**, 174-192 (2015). https://doi.org/10.1016/j.brainres.2015.04.044

10 Lake, E. M. R. *et al.* Neurovascular unit remodelling in the subacute stage of stroke recovery. *Neuroimage* **146**, 869-882 (2017). https://doi.org/10.1016/j.neuroimage.2016.09.016

11 Crouzet, C. *et al.* Cerebral blood flow is decoupled from blood pressure and linked to eeg bursting after resuscitation from cardiac arrest. *Biomed Opt Express* **7**, 4660-4673 (2016). https://doi.org/10.1364/BOE.7.004660

12 Shen, Y. *et al.* Quantification of cerebral vascular autoregulation immediately following resuscitation from cardiac arrest. *Ann Biomed Eng* **51**, 1847-1858 (2023). https://doi.org/10.1007/s10439-023-03210-4

13 Kirschen, M. P. *et al.* The association between early impairment in cerebral autoregulation and outcome in a pediatric swine model of cardiac arrest. *Resusc Plus* **4**, 100051 (2020). https://doi.org/10.1016/j.resplu.2020.100051

14 Schwarzmaier, S. M., Kim, S. W., Trabold, R. & Plesnila, N. Temporal profile of thrombogenesis in the cerebral microcirculation after traumatic brain injury in mice. *J Neurotrauma* **27**, 121-130 (2010). https://doi.org/10.1089/neu.2009.1114

15 Pernici, C. D. *et al.* Longitudinal optical imaging technique to visualize progressive axonal damage after brain injury in mice reveals responses to different minocycline treatments. *Sci Rep* **10**, 7815 (2020). https://doi.org/10.1038/s41598-020-64783-x
16 Obrenovitch, T. P., Chen, S. & Farkas, E. Simultaneous, live imaging of cortical spreading depression and associated cerebral blood flow changes, by combining voltage-sensitive dye and laser speckle contrast methods. *Neuroimage* **45**, 68-74 (2009). https://doi.org/10.1016/j.neuroimage.2008.11.025
17 van Beek, A. H., Lagro, J., Olde-Rikkert, M. G., Zhang, R. & Claassen, J. A. Oscillations in cerebral blood flow and cortical oxygenation in alzheimer's disease. *Neurobiol Aging* **33**, 428 e421-431 (2012). https://doi.org/10.1016/j.neurobiolaging.2010.11.016
18 Daniela, M., Grigoras, C., Cuciureanu, D. & Constantinescu, V. The circadian rhythm of arterial blood pressure in alzheimer's disease and vascular dementia. *Acta Neurol Belg* **123**, 129-137 (2023). https://doi.org/10.1007/s13760-021-01664-8
19 Oliver, P. L. *et al.* Disrupted circadian rhythms in a mouse model of schizophrenia. *Curr Biol* **22**, 314-319 (2012). https://doi.org/10.1016/j.cub.2011.12.051
20 Ghosh, K. K. *et al.* Miniaturized integration of a fluorescence microscope. *Nat Methods* **8**, 871-878 (2011). https://doi.org/10.1038/nmeth.1694
21 Miao, P., Lu, H., Liu, Q., Li, Y. & Tong, S. Laser speckle contrast imaging of cerebral blood flow in freely moving animals. *J Biomed Opt* **16**, 090502 (2011). https://doi.org/10.1117/1.3625231
22 Liu, R. *et al.* Extendable, miniaturized multi-modal optical imaging system: Cortical hemodynamic observation in freely moving animals. *Opt Express* **21**, 1911-1924 (2013). https://doi.org/10.1364/OE.21.001911
23 Cai, D. J. *et al.* A shared neural ensemble links distinct contextual memories encoded close in time. *Nature* **534**, 115-118 (2016). https://doi.org/10.1038/nature17955
24 Liberti, W. A., Perkins, L. N., Leman, D. P. & Gardner, T. J. An open source, wireless capable miniature microscope system. *J Neural Eng* **14**, 045001 (2017). https://doi.org/10.1088/1741-2552/aa6806
25 Jacob, A. D. *et al.* A compact head-mounted endoscope for in vivo calcium imaging in freely behaving mice. *Curr Protoc Neurosci* **84**, e51 (2018). https://doi.org/10.1002/cpns.51
26 Skocek, O. *et al.* Author correction: High-speed volumetric imaging of neuronal activity in freely moving rodents. *Nat Methods* **15**, 469 (2018). https://doi.org/10.1038/s41592-018-0034-y
27 Barbera, G., Liang, B., Zhang, L. F., Li, Y. & Lin, D. T. A wireless miniscope for deep brain imaging in freely moving mice. *J Neurosci Methods* **323**, 56-60 (2019). https://doi.org/10.1016/j.jneumeth.2019.05.008
28 Yanny, K. *et al.* Miniscope3d: Optimized single-shot miniature 3d fluorescence microscopy *Light-Sci Appl* **9**, 171 (2023). https://doi.org/10.1038/s41377-020-00403-7
29 Shuman, T. *et al.* Breakdown of spatial coding and interneuron synchronization in epileptic mice. *Nat Neurosci* **23**, 229-238 (2020). https://doi.org/10.1038/s41593-019-0559-0
30 Juneau, J. *et al.* Minifast: A sensitive and fast miniaturized microscope for in vivo neural recording. *bioRxiv*, 2020.2011.2003.367466 (2020). https://doi.org/10.1101/2020.11.03.367466
31 de Groot, A. *et al.* Ninscope, a versatile miniscope for multi-region circuit investigations. *Elife* **9**, e49987 (2020). https://doi.org/10.7554/eLife.49987
32 Scherrer, J. R., Lynch, G. F., Zhang, J. J. & Fee, M. S. An optical design enabling lightweight and large field-of-view head-mounted microscopes. *Nat Methods* **20**, 546-549 (2023). https://doi.org/10.1038/s41592-023-01806-1
33 Rynes, M. L. *et al.* Miniaturized head-mounted microscope for whole-cortex mesoscale imaging in freely behaving mice. *Nat Methods* **18**, 417-425 (2021). https://doi.org/10.1038/s41592-021-01104-8
34 Zong, W. *et al.* Miniature two-photon microscopy for enlarged field-of-view, multi-plane and long-term brain imaging. *Nat Methods* **18**, 46-49 (2021). https://doi.org/10.1038/s41592-020-01024-z
35 Zong, W. *et al.* Large-scale two-photon calcium imaging in freely moving mice. *Cell* **185**, 1240-1256 e1230 (2022). https://doi.org/10.1016/j.cell.2022.02.017
36 Supekar, O. D. *et al.* Miniature structured illumination microscope for in vivo 3d imaging of brain structures with optical sectioning. *Biomed Opt Express* **13**, 2530-2541 (2022). https://doi.org/10.1364/BOE.449533
37 Zhao, C. *et al.* Miniature three-photon microscopy maximized for scattered fluorescence collection. *Nat Methods* **20**, 617-622 (2023). https://doi.org/10.1038/s41592-023-01777-3

38 Guo, C. *et al.* Miniscope-lfov: A large-field-of-view, single-cell-resolution, miniature microscope for wired and wire-free imaging of neural dynamics in freely behaving animals. *Sci Adv* **9**, eadg3918 (2023). https://doi.org/10.1126/sciadv.adg3918
39 Zhang, Y. *et al.* A miniaturized mesoscope for the large-scale single-neuron-resolved imaging of neuronal activity in freely behaving mice. *Nat Biomed Eng* **8**, 754-774 (2024). https://doi.org/10.1038/s41551-024-01226-2
40 Madruga, B. A. *et al.* Open-source, high performance miniature 2-photon microscopy systems for freely behaving animals. *Nat Commun* **16**, 7125 (2025). https://doi.org/10.1038/s41467-025-62534-y
41 Dong, Z. *et al.* Simultaneous two-color imaging with a dual-channel miniscope in freely behaving mice. *Sci Adv* **11**, eadr6470 (2025). https://doi.org/DOI: 10.1126/sciadv.adr6470
42 Senarathna, J. *et al.* A miniature multi-contrast microscope for functional imaging in freely behaving animals. *Nat Commun* **10**, 99 (2019). https://doi.org/10.1038/s41467-018-07926-z
43 Podgorski, K. & Ranganathan, G. Brain heating induced by near-infrared lasers during multiphoton microscopy. *J Neurophysiol* **116**, 1012-1023 (2016). https://doi.org/10.1152/jn.00275.2016
